# Indole-acetaldehyde from *Rothia mucilaginosa* activates the PXR/NRF2 axis to enhance alveolar macrophage phagocytosis and protect against ARDS

**DOI:** 10.1186/s12931-026-03551-3

**Published:** 2026-02-19

**Authors:** Wensi Fan, Tingting Tan, Chujun Yang, Yongmei Cao, Cui Jin, Xiaohao Liu, Kangni Shang, Junjie Wang, Jingjing Xu, Yingchuan Li

**Affiliations:** 1 Department of Critical Care Medicine, Shanghai Tenth People’s Hospital, Tongji University School of Medicine, 301# Yanchang Middle Road, Jing`an District, Shanghai, 200072 People’s Republic of China; 2 Key Laboratory of Pathogen-Host Interaction, Ministry of Education, Shanghai, China

**Keywords:** *Rothia mucilaginosa*, Resident alveolar macrophage, Phagocytosis, Pregnane X receptor, Indole-3-acetaldehyde

## Abstract

**Background:**

Despite advances in therapeutic strategies, acute respiratory distress syndrome (ARDS) mortality remains high. Growing evidence links respiratory microbiome composition to ARDS outcomes. This investigation sought to elucidate how colonizing bacteria and their metabolites influence ARDS pathogenesis.

**Methods:**

Bronchoalveolar lavage fluid (BALF) from patients with pulmonary infections was analyzed by metagenomic next-generation sequencing (mNGS) to identify characteristic bacteria. Bacterial culture supernatants were analyzed by untargeted metabolomics (LC-MS) to identify metabolites. A murine ARDS model was established through intratracheal LPS instillation. Single-cell sequencing datasets from the GEO database were analyzed to reveal differential cell populations and functional alterations in murine ARDS. Potential molecular mechanisms were explored through molecular docking, RNA-seq analysis, Western boltting, and targeted gene knockdown in murine and cellular model.

**Results:**

*R. mucilaginosa* demonstrated enrichment in patients without ARDS (nARDS). The bacterial culture supernatant conferred substantial protection in murine models, whereas viable bacteria showed minimal efficacy. LC-MS analysis identified indole-3-acetaldehyde (IAAld) as the predominant metabolite in the supernatant. Single-cell sequencing suggested that resident alveolar macrophages (RAMs) were pivotal cells in murine ARDS model. IAAld enhanced RAMs phagocytosis, facilitating neutrophil and LPS clearance. Mechanistic studies revealed that IAAld likely activated PXR signaling, promoted NRF2 nuclear translocation, and upregulated the phagocytosis-related gene *CD36*. Targeted PXR knockdown eliminated these protective effects.

**Conclusion:**

The respiratory commensal *R. mucilaginosa* synthesizes IAAld, which—independent of bacterial colonization per se—ameliorates ARDS through PXR/NRF2/CD36 axis activation, thereby enhancing macrophage phagocytic function. These findings suggest that therapeutic targeting of microbial metabolites represents a novel ARDS treatment paradigm.

**Supplementary Information:**

The online version contains supplementary material available at 10.1186/s12931-026-03551-3.

## Introduction

Acute Respiratory Distress Syndrome (ARDS) is a form of respiratory failure characterized by acute hypoxemia, which can be triggered by both pulmonary and extra-pulmonary risk factors. ARDS accounts for approximately 10% of all patients in ICU. Despite the widespread application of comprehensive treatment methods, including mechanical ventilation, the mortality rate for critically ill ARDS patients remains as high as 45% [1–4]. This suggests that new perspectives are needed to explore novel mechanisms and therapeutic approaches.

Recent studies have indicated that the respiratory microbiome plays a role in the progression and prognosis of ARDS [5–7]. The widespread use of metagenomic next-generation sequencing (mNGS) in pathogen detection has significantly expanded our understanding of the respiratory microbiome. In healthy adults, the lung microbiome is predominantly composed of Firmicutes (including *Streptococcus spp.* and *Veillonella spp.*) and Bacteroidetes (including *Prevotella spp.*) [8]. Disruptions in microbial homeostasis, along with the absence of specific colonizing bacteria, have been linked to the development of ARDS [9, 10]. *Rothia mucilaginosa* (*R. mucilaginosa*) represents a distinctive respiratory tract colonizer among these commensal organisms. Previous studies have shown that *R. mucilaginosa*, an oral commensal bacterium, is enriched in patients with chronic respiratory diseases. In an adult cohort with bronchiectasis, the abundance of *R. mucilaginosa* was negatively correlated with pro-inflammatory markers [11]. While these observations suggest anti-inflammatory properties of *R. mucilaginosa*, the specific bioactive mediators—particularly bacterial metabolites—underlying this protection remain enigmatic.

Microbial communities, once colonized, can modulate the immune microenvironment through the metabolites they produce locally, offering therapeutic potential for various acute and chronic inflammatory conditions, including ARDS [12–14]. The pathophysiology of ARDS is characterized by dysregulated inflammatory responses and excessive release of pro-inflammatory cytokines [15]. This process involves the sustained recruitment of polymorphonuclear cells (PMNs), including neutrophils. While neutrophils play a crucial role in pathogen clearance, excessive or delayed clearance of PMNs leads to persistent inflammation, which is a primary cause of ARDS [16, 17]. Resident alveolar macrophages (RAMs) are critical in the resolution of inflammation by clearing PMNs [18]; however, during ARDS, macrophage phagocytic capacity is impaired, and macrophage depletion occurs, contributing significantly to the prolonged inflammatory state. The metabolite-driven anti-inflammatory mechanisms may involve direct suppression of TLR4/NF-κB signaling pathways [13], modulation of the inflammatory cytokine storm induced by excessive neutrophils aggregation, or the generation of short-chain fatty acids with anti-inflammatory properties that regulate systemic inflammation [12]. Therefore, we hypothesize that the metabolic products of *R. mucilaginosa* may exert potential anti-inflammatory effects, involving the modulation of RAMs function and the clearance of excessive neutrophils.

Tryptophan-derived compounds, including indole-3-acetaldehyde (IAAld), constitute critical microbiota metabolic outputs. These metabolites have shown regulatory effects in various acute and chronic inflammatory disease models [19, 20]. Whether *R. mucilaginosa* produces these indole derivatives to modulate RAMs in ARDS, and the associated mechanistic pathways, remains unexplored. Indole derivatives are also known to regulate immune cell behavior. For example, indole-3-propionic acid (IPA) and indole-3-acetic acid (IAA) can modulate the polarization state of monocyte-derived macrophages, promoting their shift toward an anti-inflammatory phenotype [21, 22]. Indoles exert their effects through multiple regulatory receptors, including pregnane X receptor (PXR). PXR is a nuclear receptor that regulates the induction of drug-metabolizing enzymes and transporters by xenobiotics. Although PXR is predominantly expressed in the liver and gastrointestinal tract, recent studies have demonstrated its presence in immune cells, including T and B lymphocytes [23, 24]. Recently research reported that PXR is expressed in macrophages and contributes to macrophage polarization, with PXR agonists showing promising therapeutic effects in endotoxin-induced liver injury [25]. Nuclear factor erythroid 2–related factor 2 (NRF2) is another key regulator that has been extensively studied in the context of oxidative stress and macrophage function [26]. Notably, PXR and NRF2 share overlapping downstream targets involved in redox reactions and the metabolism of endogenous and xenobiotic compounds [27, 28]. Collectively, this investigation seeks to determine whether *R. mucilaginosa*-derived IAAld modulates RAM function in ARDS via the PXR/NRF2 axis, while elucidating the underlying mechanisms.

## Materials and methods

### Patient information and mNGS analysis

Clinical data were collected from patients with pulmonary infection admitted to Shanghai Tenth People’s Hospital (2022 ~ 2023). Patients were categorized into ARDS, non-ARDS (nARDS), and ARDS death (dARDS) groups according to the 2023 Berlin definition [4]. Bronchoalveolar lavage fluid (BALF) was collected within 48 h of admission. Metagenomic next-generation sequencing (mNGS) was performed using the IDseq Ultra technology (Vision Medicals, Guangzhou, China) to detect respiratory microbiota as previously described [29].

### Bacterial culture and metabolomic profiling

*R. mucilaginosa* (ATCC 25296, BeNa Culture Collection) was streaked onto blood agar plates, and single colonies were expanded in Luria-Bertani (LB) broth. Bacterial suspensions were diluted to 10^7^ and 10^8^ CFU/mL by Phosphate Buffered Saline (PBS) for murine interventions. To verify the production of indole derivatives, centrifuged culture supernatants were collected. Untargeted metabolomic analysis of the culture supernatants was performed using Liquid Chromatography-Mass Spectrometry (LC-MS/MS, APTBIO, China) on Agilent 1290/Vanquish UHPLC systems coupled with AB Triple TOF 6600 or Q Exactive platforms to identify specific metabolic products.

### Murine ARDS model and interventions

Male C57BL/6J mice (10–12 weeks) were purchased from B&K Universal Group (Shanghai, China) and used for ARDS model (intratracheal instillation of LPS, 5 mg/kg)[30]. Animal Use License number is SYXK 2021-001.

For interventions, mice received intratracheal instillations of *R. mucilaginosa* (10^7^ or 10^8^ CFU/mL), culture supernatants, heat-inactivated bacteria or IAAld (2.5 ~ 10 µg/time) at 48 h and 24 h prior to LPS intratracheal instillation model.

RAM-specific PXR knockdown was performed by airway instillation of AAV8-F4/80-PXR shRNA vectors (1.0 × 10^12^ genome copies per mouse) 4 weeks prior to interventions or LPS challenge. RAM-depletion were induced by clodronate liposomes (Yeasen Biotechnology, China, 40 µL/mouse, 3 consecutive days) through airway instillation.

### Assessment of lung injury and inflammation

Mice were sacrificed at 48 h after ARDS model. Lung injury was assessed by the wet-to-dry (W/D) weight ratio, and semi-quantitative scoring of lung histological H&E-stained based on ATS guidelines [31]. BALF was harvested for evaluating cell and protein exudation. The concentration of inflammatory cytokines were measured by multiplex immunoassay. More details of histological staining (such as DHE and immunofluorescence) and flow cytometry analysis are described in the Supplementary Materials.

### Cell culture and molecular assays

Murine RAMs (MH-S), RAW264.7, THP-1, and A549 cells were cultured in RPMI-1640 or DMEM medium with 10% fetal bovine serum. To assess the effects of IAAld on PXR downstream gene activation, cells were pretreated with 50 ~ 250 µM IAAld or vehicle (DMSO) for 24 h, followed by stimulation with 100 ng/mL LPS (or FITC–LPS) or DMSO for an additional 24 h. Additionally, siRNA transfection was used for *Pxr* knockdown in vitro.

For mechanism exploration, qRT-PCR, Western blotting, Co-Immunoprecipitation (Co-IP), Chromatin Immunoprecipitation (ChIP), and molecular docking (Autodock Vina 1.2.2) were conducted. Transcriptome sequencing (RNA-seq) was performed by LC-Bio Technologies. Detailed protocols for the mentioned above, as well as PCR primer sequences are available in the Supplementary Materials.

### Statistical analysis

Paired data were analyzed using paired t-tests. Non-normally distributed data were compared using the Wilcoxon rank-sum test. Unpaired data were analyzed using unpaired or multiple t-tests as appropriate. One-way ANOVA was applied for comparisons among multiple groups. All analyses were performed using GraphPad Prism 9 (GraphPad Software Inc., San Diego, USA) and R 4.2.2 (https://www.r-project.org). A *p* value < 0.05 was considered statistically significant.

## Results

### *R. mucilaginosa* as a characteristic colonizer in nARDS patients and ARDS survivors

We collected clinical data from patients at Shanghai Tenth People’s Hospital (2022 ~ 2023) who underwent bronchoscopy and BALF mNGS due to pulmonary infection. Patients were classified according to the 2023 Berlin criteria. Briefly, the cohort primarily included ICU-admitted ARDS patients (predominantly with severe community-acquired pneumonia) and non-ARDS patients with mild disease (mainly community-acquired pneumonia, or pulmonary infections with clear microbiological diagnosis). The inclusion workflow is shown in Fig. S1A. Of 112 patients who underwent mNGS testing, 11 were excluded, leaving 101 patients for analysis: 49 in the ARDS group and 52 in the nARDS group. Baseline characteristics are summarized in Fig. S1B.

**Fig. 1:**
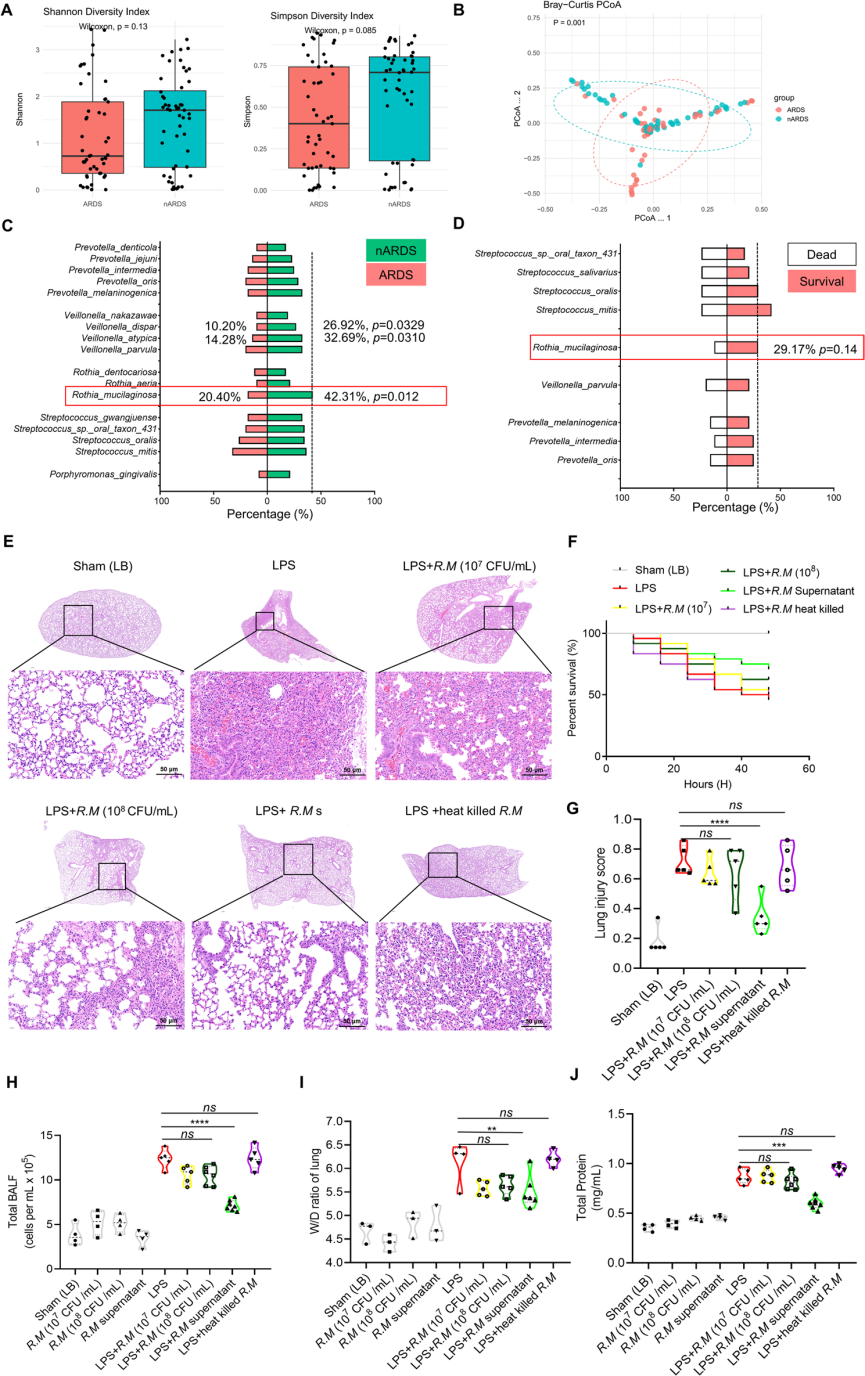
*R. mucilaginosa* exhibits increased abundance in non-ARDS lung infection and its culture supernatant alleviates LPS-induced acute lung injury in mice. **A** α-diversity analysis of the ARDS (*n* = 49) and non-ARDS (*n* = 52) groups, shown using Shannon and Simpson indices. **B** β-diversity comparison between the two groups, visualized via principal coordinate analysis (PCoA). **C** and **D** Displayed relative abundance percentages in the ARDS and non-ARDS groups, respectively. **E** Representative H&E staining results at 48 h post-modeling for each group. **F** Survival curves at 48 h following LPS-induced airway instillation in mice treated with varying concentrations of *R. mucilaginosa* supernatant or heat-killed bacterial pellets. **G** Histological damage scores, *n* = 5 random fields per group. **H** Total cell counts in bronchoalveolar lavage fluid (BALF), non-model group (*n* = 4), and LPS-model group (*n* = 5 ~ 7 per group). **I** Lung wet-to-dry weight ratio, non-model group (*n* = 3), model group (*n* = 4 ~ 6); each data point represents one mouse. **J** Protein quantification in BALF by BCA assay. Data are presented as mean ± SEM. **p < 0.01, ***p < 0.001, ****p < 0.0001, *ns*: not significant

Microbial community analysis revealed that airway microbiome α-diversity was reduced in ARDS compared with nARDS patients, as reflected by decreased Shannon index (*p* = 0.13) and Simpson index (*p* = 0.085) (Fig. 1A). Moreover, β-diversity differed significantly between groups (*p* = 0.001) (Fig. 1B). Similarly, ARDS non-survivors displayed lower α-diversity compared with survivors; both α- and β-diversity comparisons are shown in Fig. S1C, D.

Importantly, *R. mucilaginosa* emerged as a characteristic colonizer with higher relative abundance in the nARDS group compared with ARDS patients. Specifically, the relative abundance of *R. mucilaginosa* was 42.31% in the nARDS group (*p* = 0.012). Although the difference between ARDS survivors (29.17%) and non-survivors did not reach statistical significance (*p* = 0.14) (Fig. 1C, D), the presence of *R. mucilaginosa* in survivors hinted at a potential benefit, prompting us to investigate whether its functional activity, rather than mere abundance, contributes to protection.

### *R. mucilaginosa* and its supernatant alleviate LPS-induced ARDS in mice

To evaluate the role of *R. mucilaginosa*, we established an LPS-induced ARDS mouse model via intratracheal instillation (5 mg/kg). Mice were pretreated with live *R. mucilaginosa* at concentrations of 10^7^ or 10^8^ CFU/mL, the culture supernatant of *R. mucilaginosa* (10^8^ CFU/mL), or heat-inactivated bacterial components at 48 h, 24 h, and immediately prior to LPS administration. Corresponding control groups received the same interventions but without subsequent LPS challenge.

Although treatment with 10^8^ CFU/mL *R. mucilaginosa* modestly reduced histopathological lung injury (Fig. 1E and G), decreased cellular infiltration and protein levels in BALF (Fig. 1H and J), lowered the lung wet-to-dry weight ratio (Fig. 1I), and improved survival (Fig. 1F), these effects did not reach statistical significance. In contrast, the culture supernatant of *R. mucilaginosa* exhibited the most pronounced protective effect across these parameters. Notably, heat inactivation completely abolished the protective effect, indicating that the benefit was dependent on metabolically active products rather than bacterial structural components.

Together, these findings suggest that the potential protective role of *R. mucilaginosa* in ARDS stems primarily from secreted metabolites rather than bacterial cells per se. This observation clarifies why protection might not correlate strictly with bacterial abundance, as metabolic output exhibits considerable variability. To identify the responsible bioactive components, we subsequently characterized the metabolic products of *R. mucilaginosa*.

### Indole derivatives are the dominant metabolites in *R. mucilaginosa* supernatant and exert protective effects in ARDS

**Fig. 2:**
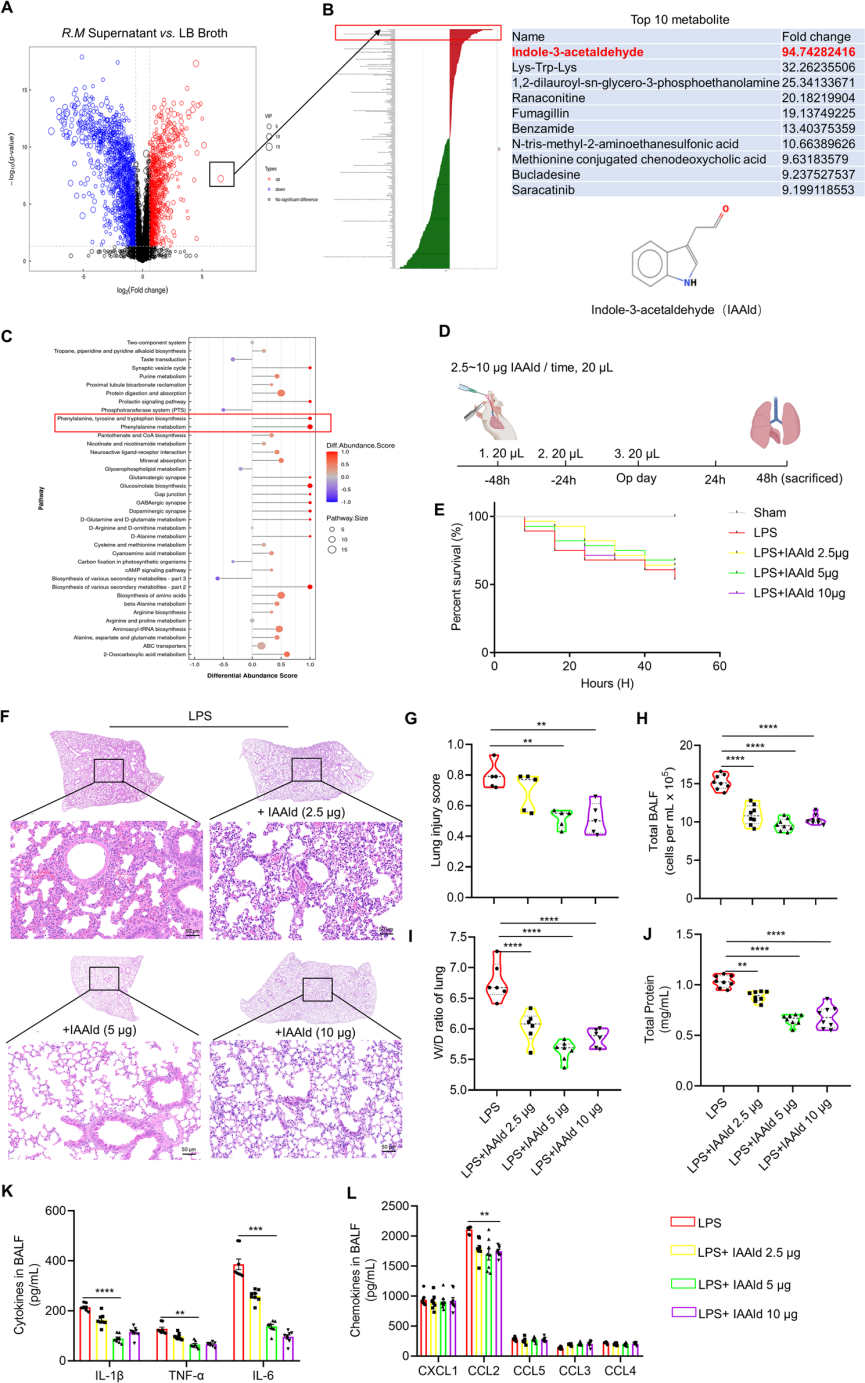
The dominant metabolic product of *R. mucilaginosa* supernatant is indole-3-aldehyde (IAAld), which exerts protective effects in ARDS mice. **A** Non-targeted metabolomics analysis of supernatant compounds (LB medium *vs. R. mucilaginosa* supernatant), with volcano plot displaying Log2 fold change (FC) and Variable Importance in Projection (VIP) scores used to identify the dominant metabolite, IAAld. **B** Visualization of LogFC for IAAld, ranked first in the analysis. IAAld’s chemical formula was obtained through PubChem. **C** Differential abundance scores (DA score) for metabolic pathways associated with IAAld. **D** Experimental timeline: IAAld (2.5 µg ~ 10 µg) was administered 48 h, 24 h, and on the day of modeling. The control group received the same volume of DMSO (20 µL) as a solvent for IAAld, with LPS (5 mg/kg) aerosol instillation for modeling. **E** Survival curves for each group after modeling and intervention. **F** Representative lung histological sections. **G** Histological damage scores, *n* = 5. **H** Cell counts in bronchoalveolar lavage fluid (BALF), *n* = 8. **I** Lung wet-to-dry weight ratio, *n* = 6 ~ 7. **J** BCA assay protein quantification in supernatant, *n* = 8, Cytokines and chemokines were analyzed by multiplex flow cytometry (**K** and **L**), *n* = 8. Data are presented as mean ± SEM. **p < 0.01, ***p < 0.001, ****p < 0.0001

To identify characteristic metabolites and confirm their bacterial origin, we performed untargeted metabolomic profiling of the bacteria-only culture supernatant (in the absence of host cells). Based on log2 fold change (FC) and variable importance in projection (VIP) values, we found that tryptophan-derived indole metabolites—specifically IAAld and indole-3-butyric acid—were enriched at high levels (Fig. 2A, B; Fig. S2A). The detection of IAAld in this isolated culture system provided direct evidence that *R. mucilaginosa* possesses the enzymatic machinery to synthesize IAAld from tryptophan. Further pathway enrichment analysis using difference abundance scores (DA scores) demonstrated significant enrichment of the “phenylalanine, tyrosine, and tryptophan biosynthesis” and “phenylalanine metabolism” pathways (Fig. 2C), suggesting that indole derivatives represent the dominant metabolic products of *R. mucilaginosa*. Based on these findings, we selected IAAld for further functional evaluation in ARDS.

To quantify IAAld concentration in the supernatant and establish an intervention dosage for subsequent experiments, we measured its ultraviolet absorption spectrum. IAAld displayed a characteristic peak at 470 nm (Fig. S2E). Using this wavelength, we constructed a standard calibration curve with known IAAld concentrations, demonstrating a strong linear correlation between absorbance and concentration (R² = 0.9976; Fig. S2F). Applying this calibration, we determined that each intervention dose of *R. mucilaginosa* supernatant contained approximately 5 µg of IAAld. Based on this, we administered 2.5 µg, 5 µg, and 10 µg doses of IAAld in the murine ARDS model, following the same intervention schedule as before (Fig. 2D). All doses alleviated ARDS manifestations, particularly 5 µg and 10 µg, as evidenced by reduced pulmonary exudation (Fig. 2H–J), mitigation of histological injury (Fig. 2F and G), and improved survival rates (Fig. 2E). For subsequent experiments, the median dose of 5 µg was selected.

We further assessed the effect of IAAld on pulmonary inflammatory mediators and chemokines. Notably, pro-inflammatory cytokines including IL-1β, TNF-α, and IL-6 were significantly reduced following IAAld treatment (Fig. 2K). In contrast, chemokine levels were largely unaffected, with the exception of CCL2, which showed a modest reduction (Fig. 2L).

In summary, these findings indicate that IAAld is a key protective metabolite derived from *R. mucilaginosa* culture supernatant, capable of attenuating lung inflammation and improving ARDS outcomes within a defined concentration range. Interestingly, its protective effect appears to be mediated primarily through suppression of inflammatory cytokines rather than broad modulation of chemokine expression.

### IAAld exerts protective effects in ARDS by modulating macrophage function

**Fig. 3:**
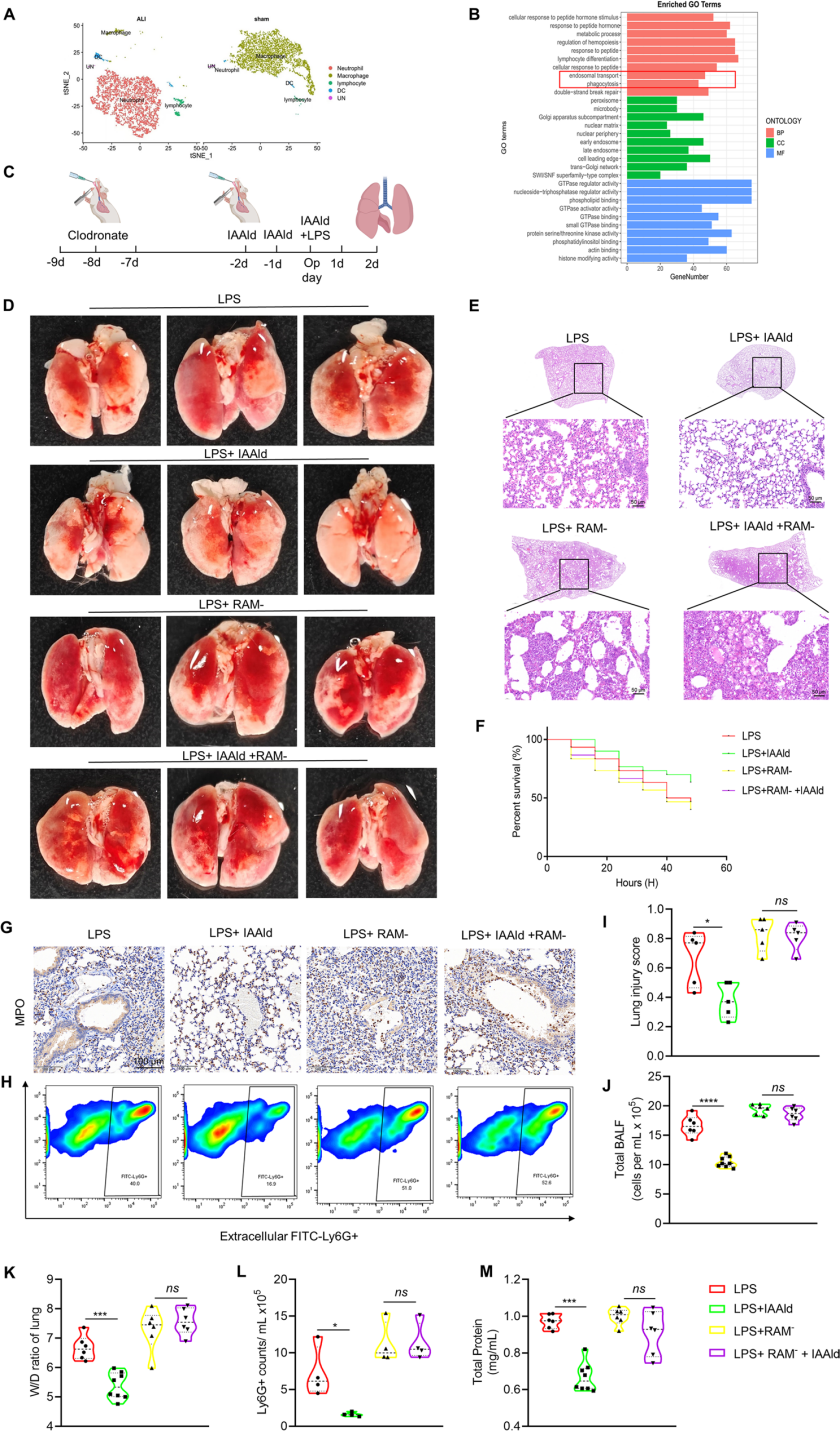
IAAld exerts regulatory effects via resident alveolar macrophages (RAMs). **A** t-SNE plot showing cell clustering changes in single-cell sequencing data from bronchoalveolar lavage fluid (BALF) of mice with acute lung injury (GSE274823, GEO). **B** Gene Ontology (GO) enrichment analysis revealing downregulated cellular functions associated with IAAld treatment. **C** Experimental timeline. **D** Representative gross images of mouse lungs. **E** Representative lung histological sections. **F** Survival curves following RAM depletion and IAAld intervention in ARDS mice. **G** Representative lung MPO immunohistochemical staining. **H** Flow cytometry analysis of extracellular Ly6G+ cells. **I** Histological damage scores, *n* = 5. **J** Cell counts in BALF, *n* = 6 ~ 8. **K** Lung wet-to-dry weight ratio, *n* = 6 ~ 7. **L** Ly6G+ (neutrophils) cell counts, detected by flow cytometry, calculated as the percentage of Ly6G+ cells * total BALF cell count, *n* = 4. **M** BCA assay protein quantification in supernatant, *n* = 6 ~ 8. Data are presented as mean ± SEM. *p < 0.05, **p < 0.01, ***p < 0.001, ****p < 0.0001, *ns*: not significant

We analyzed single-cell RNA sequencing data from BALF samples of acute lung injury (ALI) mice in the GEO database (GSE274823). Our analysis revealed a significant increase in neutrophil numbers in the BALF, accompanied by a reduction in alveolar macrophages during ALI (Fig. 3A). Gene Ontology (GO) enrichment analysis further indicated that macrophage phagocytic activity and endosomal transport functions were impaired in the ALI model (Fig. 3B).

ARDS is characterized by extensive neutrophil infiltration in the lungs, and timely clearance of these cells is crucial to minimize tissue damage and resolve inflammation. Enhanced macrophage phagocytosis of apoptotic neutrophils, cell debris, and microorganisms plays a vital role in inflammation resolution. To explore the role of macrophages in this process, we pre-treated mice with clodronate liposome aerosol to deplete RAMs. Compared to the vehicle group, clodronate liposomes significantly reduced the proportion of RAMs (Fig. S3B–D).

After RAM depletion (RAM^−^), we further investigated the effect of IAAld in the ARDS model (Fig. 3C). Our results showed that RAM^−^ negated the protective effects of IAAld (LPS + RAM^−^ vs. LPS + RAM^−^ + IAAld group), as evidenced by increased 48-hour mortality (Fig. 3F), exacerbated histological damage (Fig. 3D, E and I), and elevated lung wet/dry weight ratios (Fig. 3K). To further explore the impact on neutrophils, we measured the number and activity of neutrophils in the BALF. Using myeloperoxidase (MPO) as a marker of activated neutrophils, we found that RAM^−^ reversed the reduction in activated neutrophils caused by IAAld (Fig. 3G). Moreover, IAAld decreased the proportion of Ly6G+ neutrophils in BALF after LPS stimulation (Fig. 3H), a marker commonly used to identify neutrophils in mice. However, this effect of IAAld was lost after RAM depletion (LPS + RAM^−^ vs. LPS + RAM^−^ + IAAld) (Fig. 3L).

In a nutshell, our findings suggest that RAM plays a crucial role in mediating the protective effects of IAAld in ARDS. IAAld likely affects RAM by enhancing its phagocytic clearance of neutrophils, rather than by altering chemotaxis. Thus, we propose that RAM-mediated phagocytosis is central to IAAld’s protective function in ARDS, warranting further investigation into the macrophage-mediated immune response in the context of acute lung injury.

### IAAld activates PXR in alveolar macrophages

**Fig. 4:**
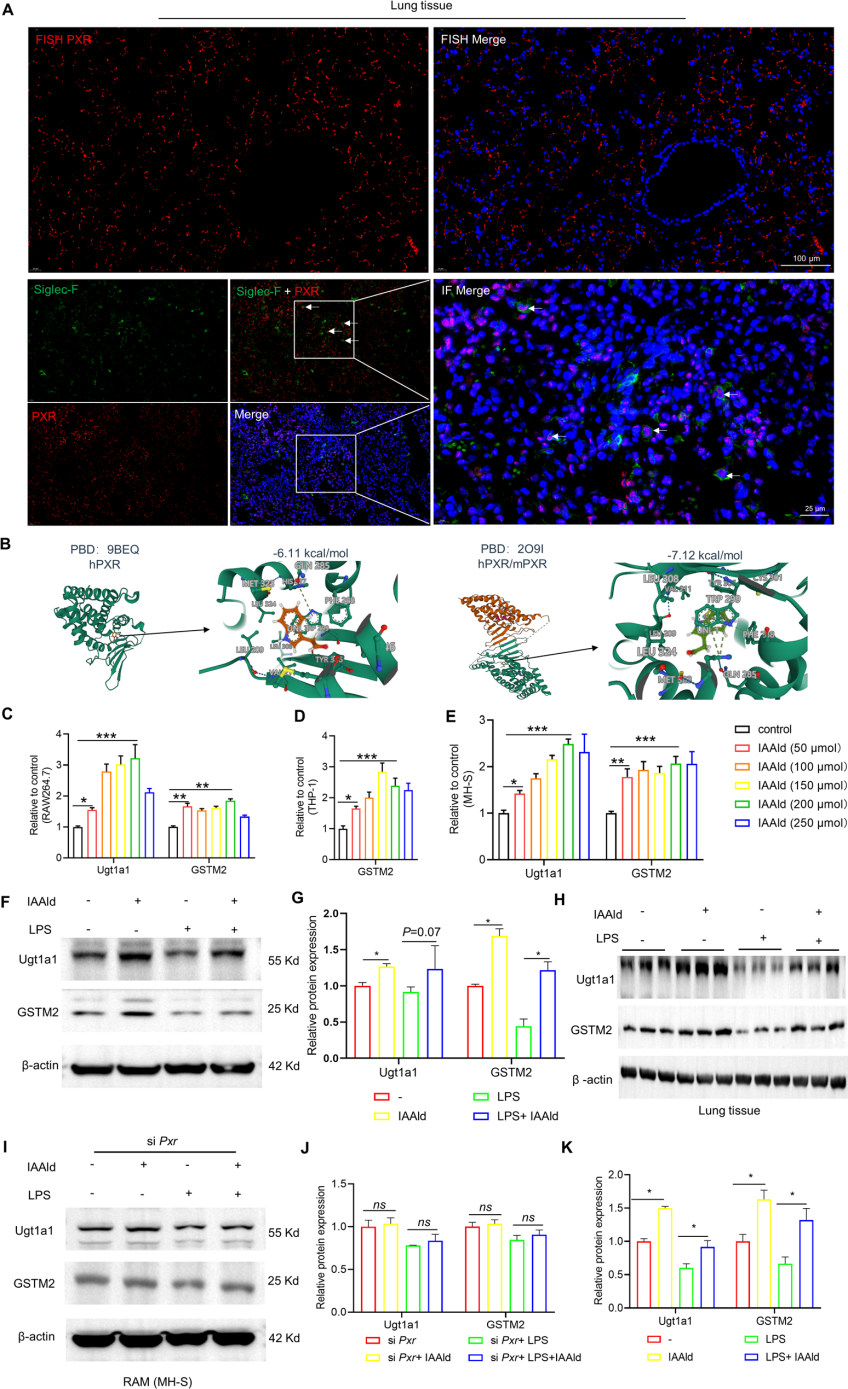
PXR receptor expression in mouse lungs and activation by IAAld in m/hPXR receptors. **A** Immunofluorescence co-staining (Siglec-F, green + PXR, red) and in situ hybridization (FISH) probes showing PXR expression in mouse lung, particularly in resident alveolar macrophages (RAMs). White arrows indicate dual-positive staining, representing RAMs expressing PXR. **B** Molecular docking simulations of PXR receptor binding, using PBD codes 9BEQ (hPXR) and 2O9I (hPXR/mPXR). **C**–**E** RT-qPCR analysis of PXR downstream gene activation in different macrophage cell lines: RAW264.7 (**C**), THP-1 (**D**), and MH-S (**E**), following IAAld treatment (*n* = 6). **F** Western blot analysis of PXR downstream proteins GSTM2 and Ugt1a1 in mouse lung tissue (*n* = 3). **G** Semi-quantitative analysis of the Western blot data in (**F**). **H**-**I** Western blot detection of GSTM2 and Ugt1a1 protein levels in lung tissue and MH-S (RAMs) cells (*n* = 3). **J**-**K** Semi-quantitative analysis of the Western blot data in (**I**) and (**H**). Data are presented as mean ± SEM. *p < 0.05, **p < 0.01, ***p < 0.001, *ns*: not significant

To further elucidate the mechanism by which IAAld modulates macrophage function, we employed SuperPred (http://www.hsls.pitt.edu)[32], which predicted PXR (Probability 82.57%, Model accuracy 94.73%) and the muscarinic acetylcholine M5 receptor (M5, Probability 86.66%, Model accuracy 94.62%) as potential receptors (Fig. S4A). Immunohistochemical staining of LPS-induced lung injury tissues revealed that, compared with PXR, M5 was barely expressed in the murine lung (Fig. S4B). Further fluorescence in situ hybridization (FISH) and immunofluorescence co-localization (SiglecF + PXR) confirmed the presence of PXR in murine lungs and specifically in RAMs (Fig. 4A).

Given that PXR display species-specific structural differences, selective agonists are often required for human (hPXR) and murine (mPXR) activation [25]. We performed molecular docking using DockMoe with hPXR (PDB ID: 9BEQ) and mPXR (PDB ID: 2O9I), revealing binding energies of -6.11 kcal/mol and − 7.12 kcal/mol, respectively (Fig. 4B). To functionally validate these findings, we examined PXR downstream gene expression (Ugt1a1, GSTM2, Cyp3a4 in human cells; Cyp3a11 in murine cells) in multiple macrophage/monocyte lines and epithelial cells. In murine RAW264.7 and MH-S cells, as well as human THP-1 cells, Cyp3a expression was minimal (CT > 35). However, IAAld treatment dose-dependently upregulated Ugt1a1 and GSTM2 expression, including in MH-S cells (a murine alveolar macrophages cell line) (Fig. 4C–E). In addition, IAAld induced Ugt1a1, GSTM2 and Cyp3a4 expression in A549 cells (Fig. S4C).

Protein-level analyses confirmed that IAAld increased Ugt1a1 and GSTM2 in murine lungs and RAM under both physiological and pathological conditions (Fig. 4F–H and K). Knockdown of PXR in RAM abrogated the ability of IAAld to induce Ugt1a1 and GSTM2, confirming PXR dependence. Interestingly, even in macrophage-depleted mice, IAAld retained the ability to increase Ugt1a1 and GSTM2 expression (Fig. S3E–F), suggesting that IAAld can also act directly on other PXR-expressing cells.

Collectively, these findings demonstrate that PXR is expressed in murine lungs and RAM, and that IAAld functions as a PXR agonist capable of activating downstream detoxification and metabolic pathways in both murine and human cells.

### PXR mediates IAAld-enhanced phagocytic function

**Fig. 5:**
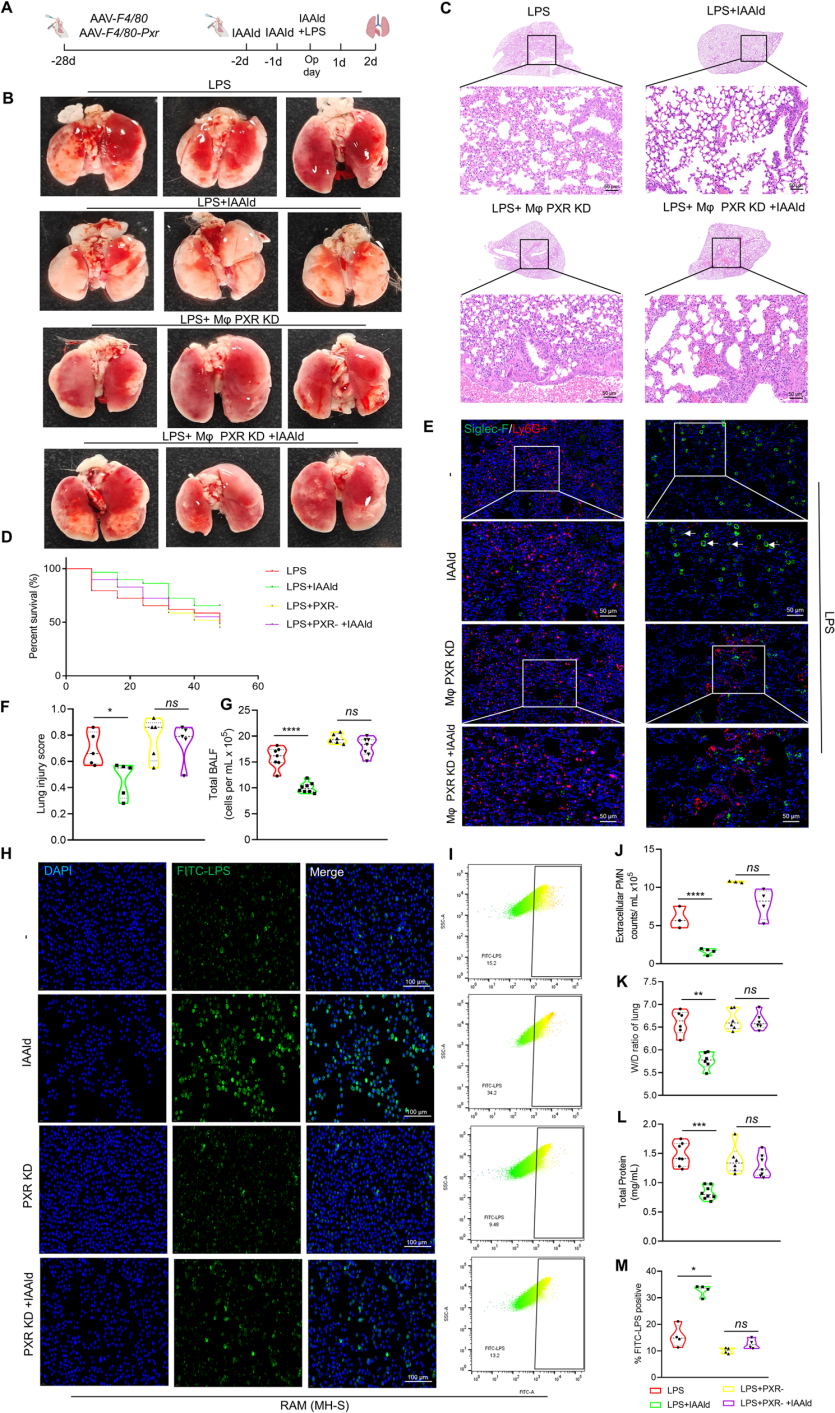
Specific inhibition of RAM PXR counteracts the protective effects of IAAld in ARDS. **A** Experimental timeline. **B** Representative gross images of mouse lungs. **C** Representative lung histological sections. **D** Survival curves for ARDS mice following RAM PXR inhibition and IAAld treatment. **E** Co-localization of Siglec-F and Ly6G to assess RAM phagocytosis of neutrophils. **F** Histological damage scores, *n* = 5. **G** Cell counts in bronchoalveolar lavage fluid (BALF), *n* = 6 ~ 8. **H** Immunofluorescence detection of RAM phagocytosis of LPS. **I** Flow cytometry analysis of FITC-LPS-positive cells. **J** Neutrophil counts, *n* = 3 ~ 4. **K** Lung wet-to-dry weight ratio, *n* = 6. **L** BCA assay protein quantification in supernatant, *n* = 6 ~ 8. **M** Quantification of data in (**I**), *n* = 4. Data are presented as mean ± SEM. *p < 0.05, **p < 0.01, ***p < 0.001, ****p < 0.0001, *ns*: not significant

To determine the role of PXR in regulating the phagocytic function of alveolar macrophages, we used an adeno-associated virus (AAV) carrying the F4/80 promoter to specifically knockdown PXR in macrophages (MφPXR KD), and subsequently evaluated the therapeutic effect of IAAld in ARDS (Fig. 5A). Four weeks after intratracheal administration of AAV-F4/80 or AAV-F4/80-pxr, PXR expression in alveolar macrophages was assessed by immunofluorescence staining of BALF and lung tissue. AAV-F4/80-pxr effectively reduced PXR expression in alveolar macrophages (Fig. S5).

In mice pretreated with AAV-F4/80-pxr, the protective effect of IAAld was abolished, as shown by aggravated histopathological injury (Fig. 5B, C and F), increased protein and cellular leakage (Fig. 5L and G), elevated wet-to-dry lung weight ratio (Fig. 5K), and higher mortality (Fig. 5D). Double staining with Siglec-F (a RAM marker) and Ly6G revealed that IAAld enhanced the clearance of PMNs by RAMs, thereby reducing neutrophil accumulation in lung tissue. However, this IAAld-induced enhancement of phagocytosis was abrogated following PXR knockdown (Fig. 5E and J).

In vitro, co-incubation of FITC-labeled LPS with MH-S confirmed these findings. IAAld significantly increased the uptake of FITC-LPS, whereas this effect was eliminated upon PXR inhibition (Fig. 5F and I). Collectively, these results indicate that IAAld enhances the phagocytic capacity of RAMs to eliminate PMNs and pro-inflammatory bacterial components, and that this effect is mediated by PXR.

### Activation of the PXR/NRF2 signaling axis during the process of macrophage phagocytosis

**Fig. 6:**
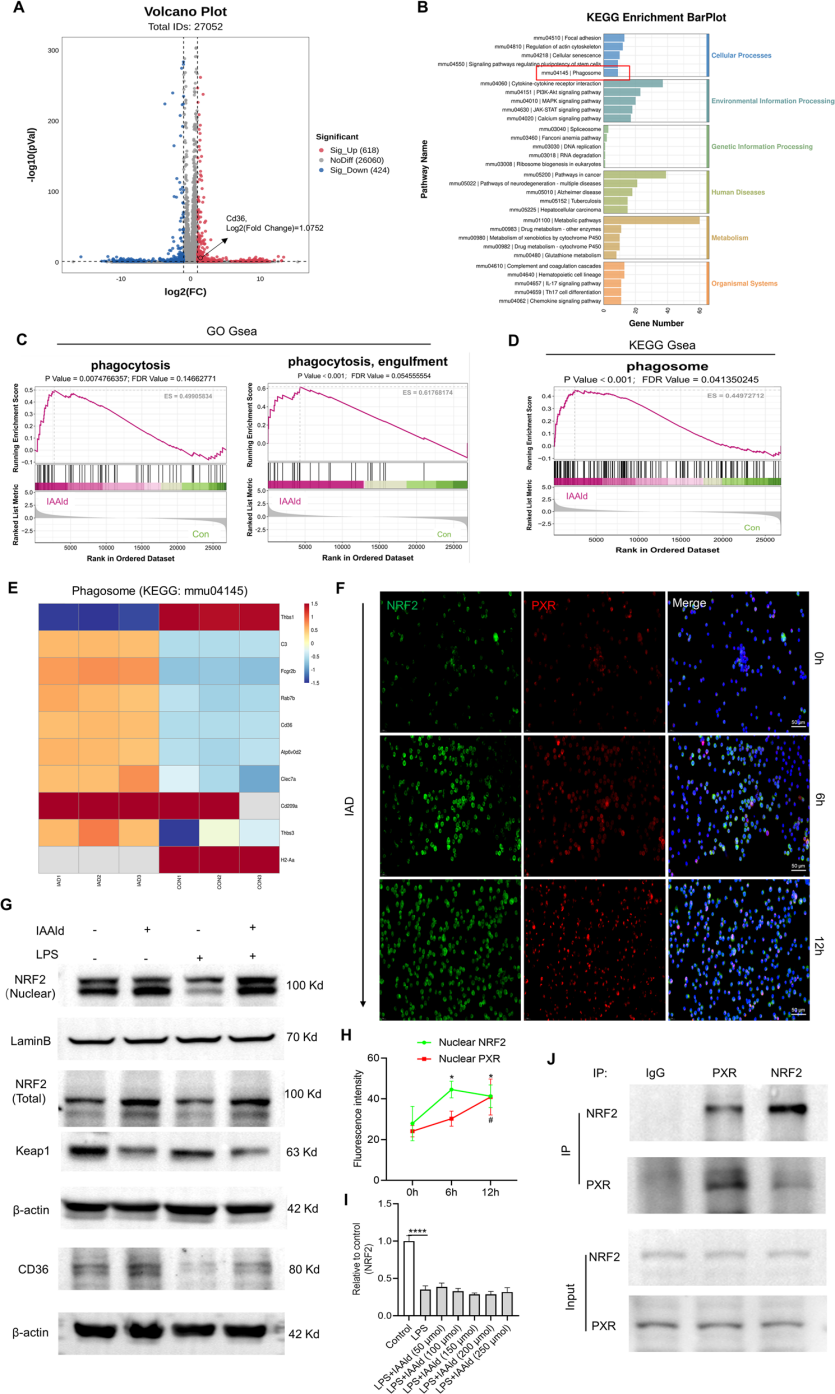
IAAld activates the PXR-NRF2-CD36 pathway. **A** Volcano plot showing differential gene expression following IAAld intervention (LPS vs. LPS+IAAld). **B** KEGG enrichment analysis identifying significantly altered cellular functions after IAAld intervention. **C** Gene Set Enrichment Analysis (GSEA) of phagocytosis-related functions using Gene Ontology (GO) database. **D** GSEA of phagocytosis-related pathways using the Kyoto Encyclopedia of Genes and Genomes (KEGG) database. **E** Heatmap of differential genes associated with “phagosome” in KEGG pathway mmu04145. **F** Immunofluorescence co-staining of NRF2 and PXR to assess the effect of IAAld on nuclear localization over time. **G** Western blot analysis of NRF2, Keap1, and CD36 protein expression following IAAld treatment under physiological conditions and LPS stimulation. **H** Semi-quantitative analysis of the Western blot data in (**G**). **I** RT-qPCR analysis of NRF2 mRNA expression levels, *n* = 3. **J** Co-immunoprecipitation (co-IP) assay demonstrating the interaction between PXR and NRF2 proteins. In (**H**), * indicates comparisons between nuclear NRF2 at 6 h and 12 h vs. 0 h (*p < 0.05); # indicates comparison between nuclear PXR at 12 h and 0 h (#*p* < 0.05). Data are presented as mean ± SEM. ****p < 0.0001

To further investigate the mechanisms underlying IAAld-mediated regulation of RAMs phagocytosis, we performed transcriptomic RNA-seq analysis comparing gene expression profiles between LPS and LPS+IAAld groups. IAAld treatment resulted in 618 upregulated and 2,060 downregulated genes, using a cutoff of log₂ FC > 1 (Fig. 6A). Kyoto Encyclopedia of Genes and Genomes (KEGG) pathway enrichment revealed significant enrichment in phagosome (mmu04145), cytokine–cytokine receptor interaction (mmu04060), and xenobiotic metabolism (mmu00983), implicating pathways associated with PXR activation (Fig. 6B). Moreover, gene set enrichment analysis (GSEA) of GO and KEGG terms demonstrated that IAAld enhanced the expression of phagocytosis-related genes (Fig. 6C, D). A heatmap of phagosome-related genes (mmu04145) highlighted the upregulation of the scavenger receptor CD36 in the IAAld group, which has been previously reported to mediate macrophage clearance of apoptotic neutrophils, erythrophagocytosis, and bacterial elimination [33–35]. Among transcription factors, peroxisome proliferator-activated receptor (PPAR)-γ and NRF2 are the most studied regulators of CD36[36, 37]. Notably, RNA-seq revealed extremely low expression of PPAR in MH-S cells compared with NRF2. Given that NRF2 is a key redox-sensitive transcription factor also involved in xenobiotic metabolism and shares downstream targets with PXR, we focused on whether PXR regulates CD36 expression through NRF2.

We observed that IAAld treatment led to time-dependent increases in nuclear localization of both PXR and NRF2 in MH-S cells, as confirmed by immunofluorescence co-localization (Fig. 6F and H). Western blotting further showed that IAAld elevated total NRF2 protein levels under both physiological and LPS-stimulated conditions, decreased Kelch-like ECH-associated protein 1 (Keap1)—a negative regulator of NRF2 ubiquitination and degradation—and enhanced CD36 expression (Fig. 6G and Fig. S6H–I). IAAld also increased nuclear NRF2 protein levels (Fig. 6G). Co-immunoprecipitation confirmed an interaction between PXR and NRF2 (Fig. 6J). Interestingly, IAAld stimulation did not alter NRF2 mRNA levels (Fig. 6I).

**Fig. 7:**
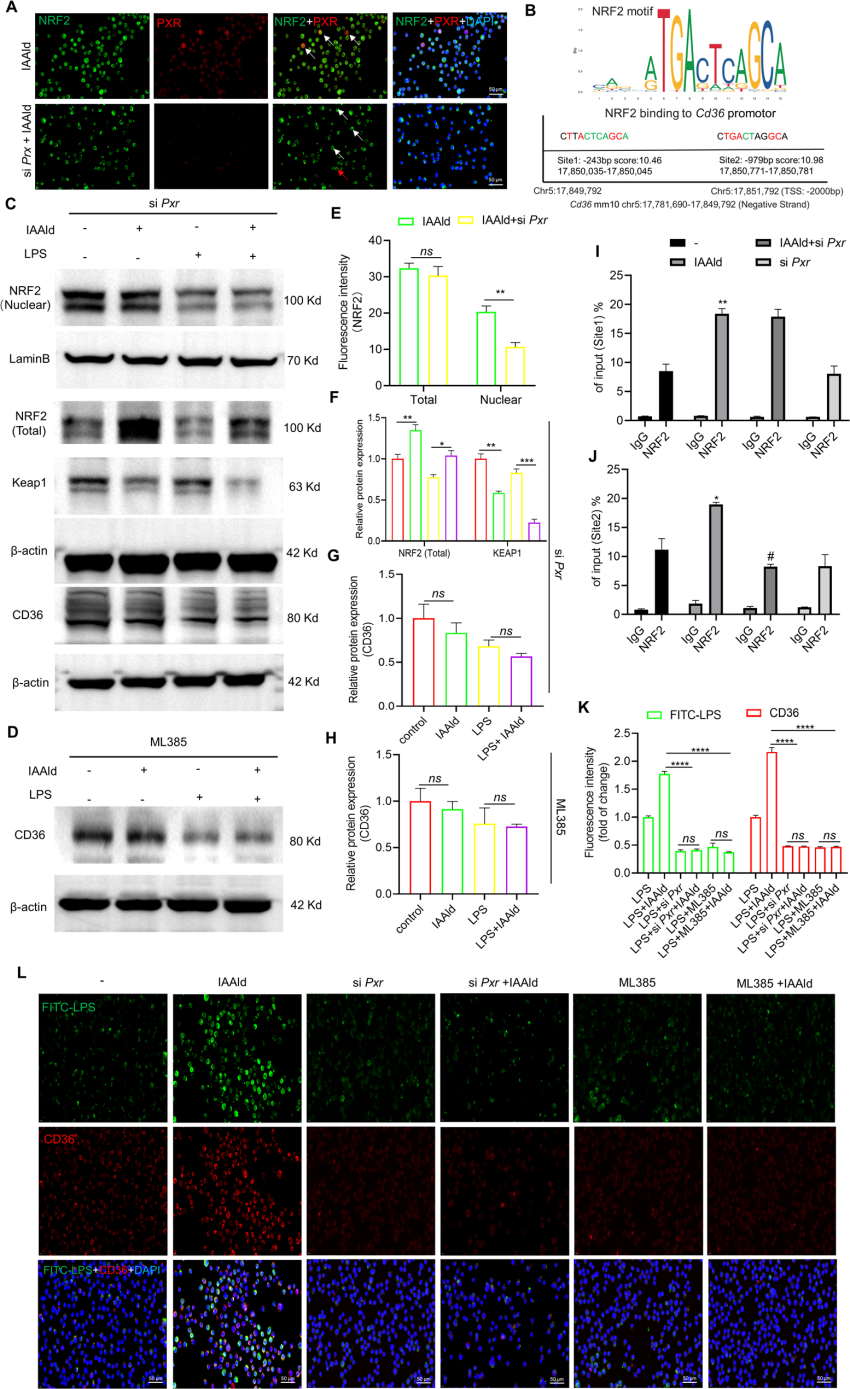
IAAld regulates phagocytosis-related gene *Cd36* via the PXR-NRF2 pathway. **A** Immunofluorescence co-localization showing the effect of PXR inhibition on NRF2 nuclear localization. White arrows indicate NRF2 localization under PXR expression or inhibition, while red arrows highlight NRF2 nuclear localization even at low PXR levels. **B** JASPAR prediction of NRF2 binding sites regulating the transcription of *Cd36*. **C** Changes in NRF2, Keap1, and CD36 protein levels following PXR inhibition. **D** Changes in CD36 protein levels following NRF2 inhibition. **E** Semi-quantitative analysis of the immunofluorescence data in (**A**), *n* = 4. **F** and (**G**) Semi-quantitative analysis of the Western blot data in (**C**). **H** Semi-quantitative analysis of the Western blot data in (**D**). **I** and (**J**) ChIP-qPCR analysis demonstrating how IAAld influences NRF2 binding to predicted *Cd36* transcriptional sites via PXR. **K** Semi-quantitative analysis of the Immunofluorescence staining data in (**L**), *n* = 4. **L** Immunofluorescence staining (CD36, red + FITC-LPS, green) to assess the effect of IAAld through the PXR-NRF2 pathway on CD36 levels and phagocytic activity. In (**I**), ** indicates comparisons between IAAld and Control group (**p < 0.01); In (**J**), * indicates comparisons between IAAld and Control group (*p < 0.05), # indicates comparison between IAAld and IAAld + si *Pxr* group (#*p* < 0.001). Data are presented as mean ± SEM. *p < 0.05, **p < 0.01, ***p < 0.001, and ****p < 0.0001, *ns*: not significant

To further investigate the relationship between PXR and NRF2, siRNA-mediated knockdown of *Pxr* was performed, and changes in NRF2 expression and subcellular localization were examined. We observed that *Pxr* knockdown markedly reduced the nuclear translocation of NRF2 (Fig. 7A, C, and E–F), while total NRF2 protein levels remained unchanged (Fig. 7E). Notably, IAAld treatment still increased total NRF2 levels and reduced Keap1 expression after *Pxr* knockdown (Fig. 7C and F), suggesting that in RAMs, IAAld regulates NRF2 nuclear translocation through a mechanism independent of the canonical NRF2/KEAP1 pathway, but potentially requiring PXR. Consistently, *Pxr* silencing attenuated NRF2-mediated transcriptional regulation of *Cd36*. To validate this observation, chromatin immunoprecipitation followed by quantitative PCR (ChIP-qPCR) was performed to examine NRF2 binding to the *Cd36* promoter under different conditions. JASPAR prediction analysis identified two putative NRF2-binding sites within the upstream region of *Cd36*: site 1 at -243 bp and site 2 at -929 bp relative to the transcription start site (TSS) (Fig. 7B). IAAld treatment enhanced NRF2 binding at both sites, whereas *Pxr* knockdown specifically reduced NRF2 occupancy at site 2. These findings suggest that IAAld-induced activation of macrophage phagocytic gene *Cd36* is mediated, at least in part, by PXR-facilitated NRF2 binding to the − 929 bp upstream region of the *Cd36* promoter (Fig. 7I, J).

To further assess whether PXR regulates macrophage phagocytosis through NRF2–CD36 signaling, we compared the effects of PXR knockdown and pharmacological inhibition of NRF2 using ML385 (5 µM). IAAld significantly increased CD36 expression and enhanced FITC-LPS phagocytosis, whereas inhibition of either PXR or NRF2 abolished this effect and even reduced phagocytic capacity (Fig. 7K, L). Finally, in vivo administration of ML385 (30 mg/kg, intraperitoneally, daily for 7 days) was performed in ARDS model. NRF2 inhibition aggravated pulmonary tissue injury and abrogated the protective effects of IAAld (Fig. S7). This was accompanied by decreased phagocytosis of PMNs (Siglec-F and Ly6G double-positive) and an increased proportion of free PMNs (Ly6G+) (Fig. S7B and G–H). Moreover, ML385 suppressed the expression of PXR downstream targets, Ugt1a1 and GSTM2, in MH-S cells (Fig. S7I–J). Collectively, these findings show that IAAld elevates NRF2 protein levels and drives its nuclear translocation in MH-S cells without altering NRF2 mRNA expression. Beyond the NRF2/KEAP1 axis, IAAld promotes NRF2 nuclear localization through PXR, which likely facilitates NRF2 nuclear import to activate CD36 transcription and enhance phagocytosis.

### IAAld alleviates oxidative stress via NRF2 pathway

As a transcription factor, NRF2 regulates antioxidant response elements (ARE), playing a central role in oxidative stress defense, which is critical in the context of ALI [38]. Using superoxide anion probes (Dihydroethidium, DHE) to assess oxidative stress levels in lung tissue, we found that IAAld significantly reduced oxidative stress following LPS stimulation. This effect was attenuated by the inhibition of RAM’s PXR (Fig. S6A and B). Additionally, IAAld increased the levels of NRF2 and ARE-regulated antioxidant proteins, such as Hmox1 and NQO1, under both physiological and pathological conditions (Fig. S6D–E). Notably, the use of AAV-F4/80-pxr did not completely reverse the increase in these proteins induced by IAAld (Fig. S6J and L). Furthermore, the downstream PXR proteins, GSTM2 and Ugt1a1, remained partly elevated in lung tissue after AAV-F4/80-pxr administration (Fig. S6J–K).

GO and GSEA enrichment analysis of RAM RNA-seq results using the keywords “oxidative stress” and “antioxidant/oxidant” revealed that only “cellular oxidant detoxification” showed statistical significance, with other enrichments lacking statistical relevance (Fig. S6C). Additional validation of MH-S cells protein levels showed no significant difference in the expression of NOQ1 and Hmox1, which are regulated by ARE, in response to IAAld (Fig. S6F–G).

These results suggest that while IAAld can reduce overall oxidative stress levels upon LPS stimulation and increase NRF2 and ARE-regulated proteins, this process likely appears to be independent of RAM.

## Discussion

ARDS remains a challenging condition with high mortality rates and a lack of disease-specific therapies [4]. Growing evidence implicates airway microbial dysbiosis in the progression of ARDS [39]. In this study, we identified *R. mucilaginosa* as a characteristic colonizer enriched in non-ARDS patients. Mechanistically, we suggest that its metabolite, IAAld, enhances alveolar macrophage phagocytosis, attenuates hyperinflammation, and improves outcomes in murine models of ARDS/ALI.

*R. mucilaginosa* is a common oral commensal frequently detected in the lower respiratory tract [40]. Previous investigations documented its inverse correlation with pro-inflammatory markers in bronchiectasis patients [11]. However, our study revealed a striking discrepancy: while live *R. mucilaginosa* airway transplantation yielded minimal therapeutic effects, bacterial culture supernatants demonstrated profound efficacy. This divergence suggests that therapeutic potential resides within active microbiome metabolites rather than bacterial presence per se. Within acute inflammatory environments, bacterial loads may prove insufficient for generating therapeutically relevant metabolite concentrations. Consequently, identifying appropriate therapeutic components becomes paramount. Furthermore, *R. mucilaginosa* has been identified as an opportunistic pathogen in multiple studies, causing fatal systemic infections [41–44]. Here, we conducted untargeted metabolomics analysis of isolated bacterial culture supernatants, identifying tryptophan metabolites with IAAld as the predominant species. IAAld detection in this bacteria-only system confirms its microbial origin. Indoles have been implicated in disease prognosis across various inflammatory conditions [21, 45]. Our investigation demonstrates that IAAld enhances the phagocytic function of RAMs, facilitating the clearance of excess neutrophils recruited during ARDS.

Excessive neutrophil infiltration is a hallmark of ARDS pathology. Furthermore, single-cell RNA sequencing of BALF also revealed depletion of RAMs, alongside weakened phagocytic activity. Selective RAM depletion eliminated IAAld’s protective effects, underscoring their indispensable role. Regarding the underlying mechanism, IAAld’s protective action likely bypasses the well-recognized Aryl Hydrocarbon Receptor (AhR) [45], as the low expression of AhR in RAMs. Instead, we identified PXR as the critical receptor. Molecular docking studies and specific PXR knockdown confirmed IAAld’s agonist activity. Notably, RAM-specific PXR knockdown negated the protective effect of IAAld in murine ARDS model, indicating that IAAld confers macrophage-dependent protection predominantly through PXR signaling.

RNA-seq further revealed that IAAld modulates macrophage phagocytosis and enriches CD36-associated pathways. Previous work have shown that established that CD36 loss exacerbates pulmonary injury [34]. Consistently, we observed IAAld-induced CD36 upregulation in RAMs. While PXR and NRF2 are recognized detoxification regulators [46, 47], their cooperative role in phagocytosis was previously unclear. We provide evidence that PXR facilitates NRF2 nuclear translocation, enabling NRF2 binding to the *Cd36* promoter for transcriptional activation. PXR depletion impaired both NRF2 nuclear translocation and CD36 expression. This mechanism may explain how IAAld simultaneously enhances phagocytosis (via CD36) and mitigates oxidative stress (via NRF2), although the antioxidant effect appeared partially dependent on RAMs.

Our study does have several limitations. First, the ARDS patient data were collected from a single center, and given the complex etiology of ARDS, larger cohort studies would provide more precise insights. Second, although we confirmed PXR activation in both murine and human cell lines, we recognize substantial species differences in PXR ligand-binding domains between mice and humans. Although IAAld demonstrated binding affinity to both species, future investigations must validate specific dosing and efficacy in human-relevant models. Third, the macrophage cell lines used in this study represent classical monocyte-derived macrophages, which may differ from primary cells. Finally, we acknowledge the dynamic chemical nature of IAAld as an intermediate metabolite. To ensure experimental precision, we utilized freshly prepared solutions for all interventions. However, given IAAld’s potential rapid turnover in complex biological matrices, quantifying its absolute accumulation in human BALF presents technical challenges. This transient property suggests that future research utilizing isotope-tracing methodologies would prove valuable for mapping metabolic flux and exploring whether downstream conversion products also contribute to ARDS protection.

**Fig. 8:**
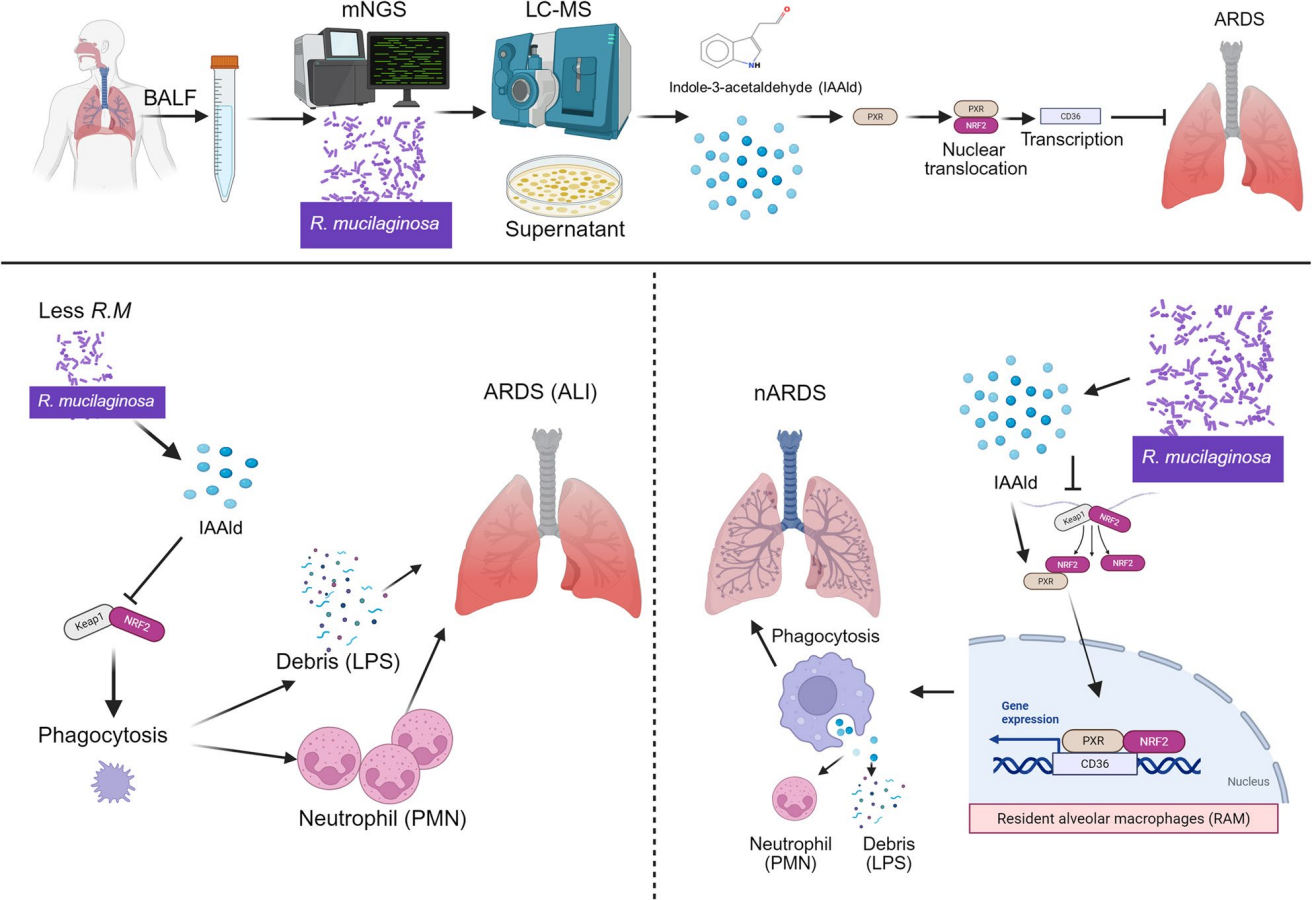
*R. mucilaginosa*–derived IAAld promotes NRF2 nuclear localization via PXR, induces CD36 expression, enhances RAM phagocytosis, and mitigates ARDS

In conclusion, our study identifies the lower respiratory tract commensal *R. mucilaginosa* as a characteristic colonizer associated with non-ARDS states. Notably, while live bacterial intervention showed limited efficacy, we discovered that its metabolite, IAAld, significantly mitigated murine ARDS. Mechanistically, our findings suggest that IAAld acts as a PXR agonist, facilitating NRF2 nuclear translocation and upregulating transcription of the phagocytosis-related gene *CD36*. This process appears to enhance the phagocytic capacity of RAMs, promoting the clearance of neutrophils and proinflammatory LPS. These results suggest that targeting microbial metabolites, specifically the IAAld-PXR axis, may represent a promising ARDS therapeutic strategy, warranting further clinical validation **(**Fig. 8**)**.

## Supplementary Information

Supplementary Material 1.

Supplementary Material 2.

Supplementary Material 3.

## Data Availability

No datasets were generated or analysed during the current study.

## Abbreviations

AAV: adeno-associated virus
AhR: Aryl Hydrocarbon Receptor
ALI: acute lung injury
ARDS: acute respiratory distress syndrome
ARE: antioxidant response elements
BALF: Bronchoalveolar lavage fluid
CFU: Colony-forming units
ChIP-qPCR: chromatin Immunoprecipitation followed by quantitative PCR
DA scores: difference abundance scores
DHE: Dihydroethidium
FC: fold change
FISH: fluorescence in situ hybridization
GO: Gene Ontology
GSEA: gene set enrichment analysis
H&E: hematoxylin and eosin
IAA: indole-3-acetic acid
IAAld: indole-3-acetaldehyde
IPA: indole-3-propionic acid
Keap1: Kelch-like ECH-associated protein 1
KEGG: Kyoto Encyclopedia of Genes and Genomes
LC-MS: Liquid Chromatography-Mass Spectrometry
LPS: Lipopolysaccharide
mNGS: metagenomic next-generation sequencing
NRF2: Nuclear factor erythroid 2–related factor 2
PBS: Phosphate Buffered Saline
PMNs: polymorphonuclear cells
PPAR-γ: proliferator-activated receptor-γ
PXR: pregnane X receptor
RAMs: resident alveolar macrophages
TSS: transcription start site
VIP: variable importance in projection
