# Supplementary material for: Indole-acetaldehyde from Rothia mucilaginosa activates the PXR/NRF2 axis to enhance alveolar macrophage phagocytosis and protect against ARDS
Source: Respir Res. 2026 Feb 19;27:112. doi: 10.1186/s12931-026-03551-3 (PMC12961788; doi:10.1186/s12931-026-03551-3)
Supplement: Supplementary file 2 — Supplementary Material 2. [file 12931_2026_3551_MOESM2_ESM.pdf]

NRF2

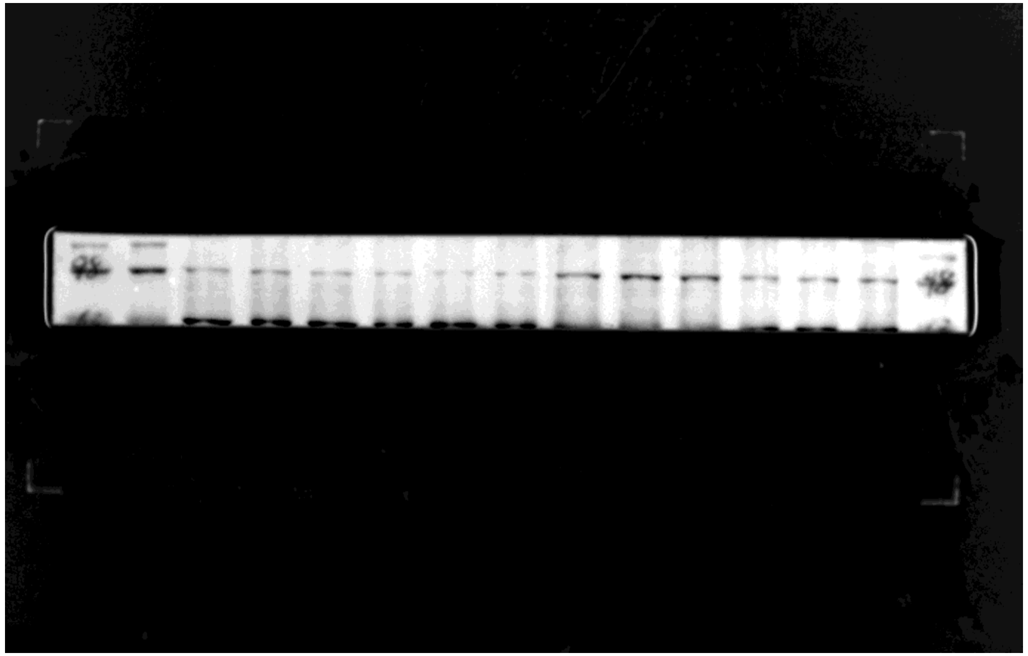

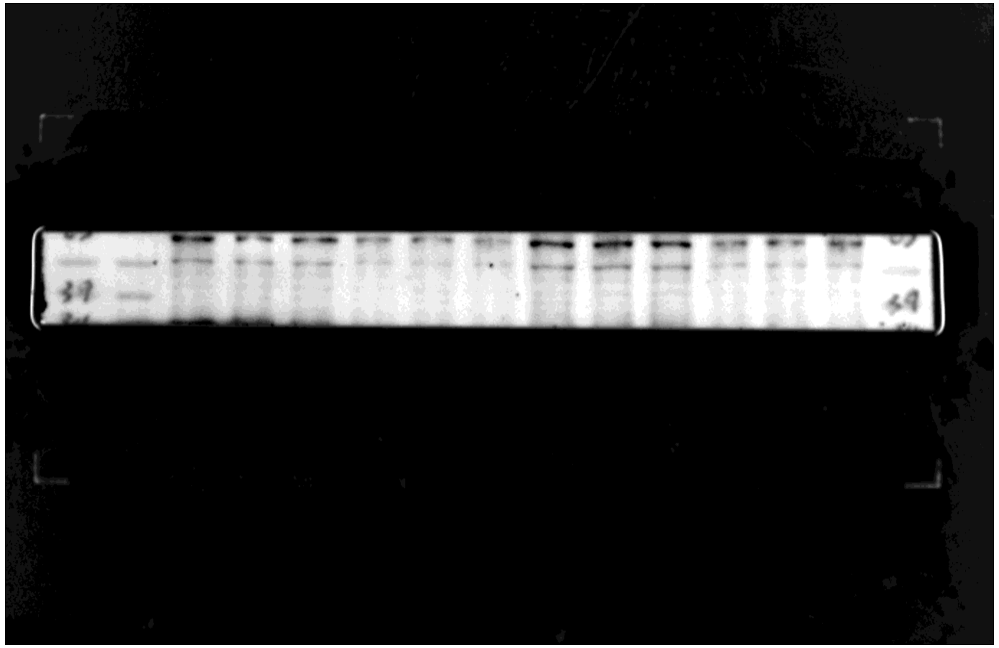

Ugt1a1

GSTM2

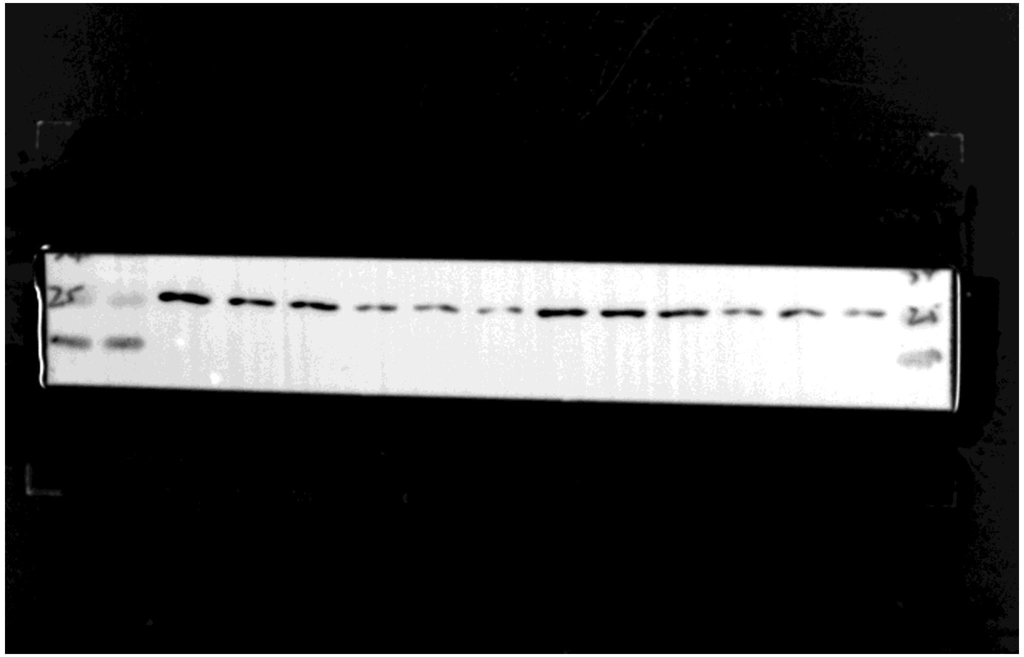

β-actin

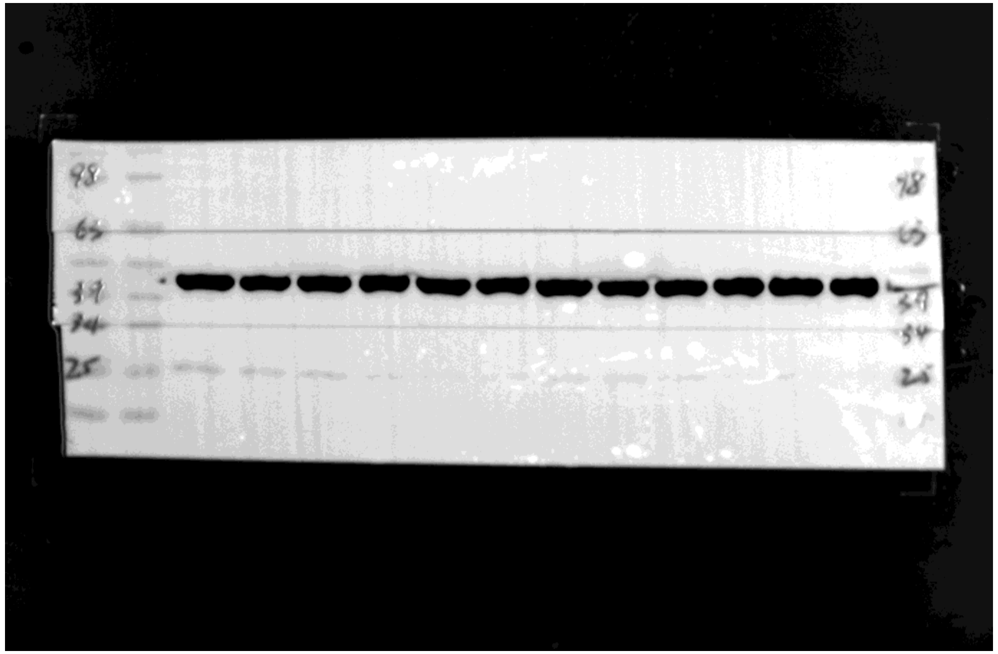

Merge

Fig.S3E

Pxr

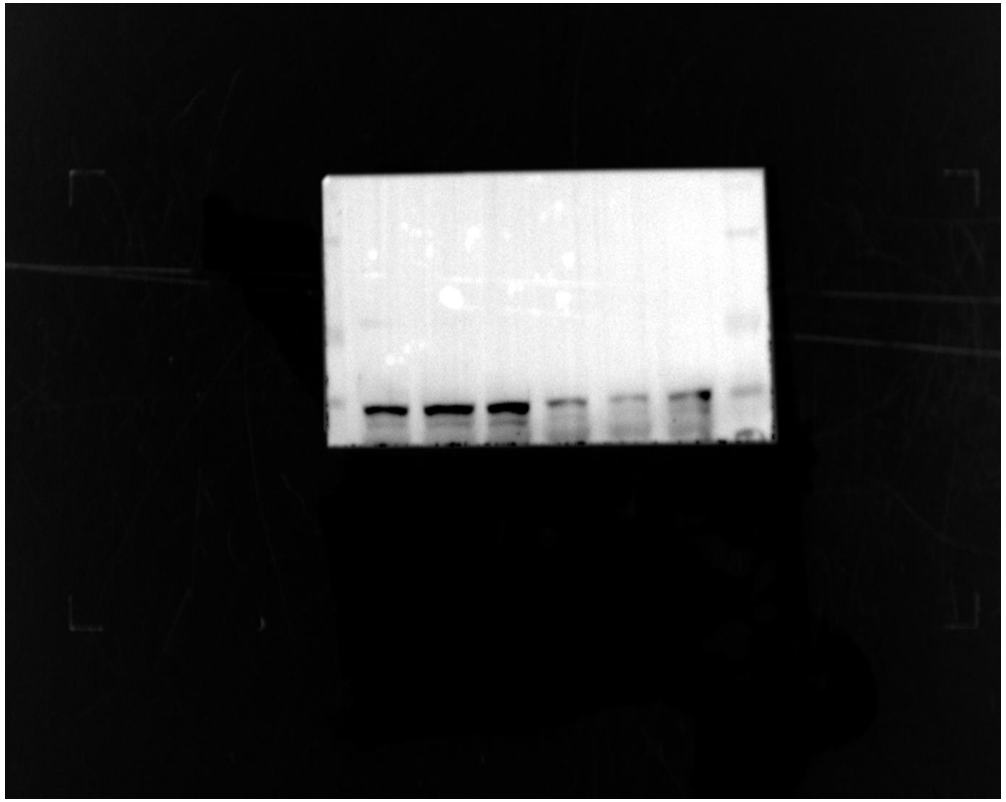

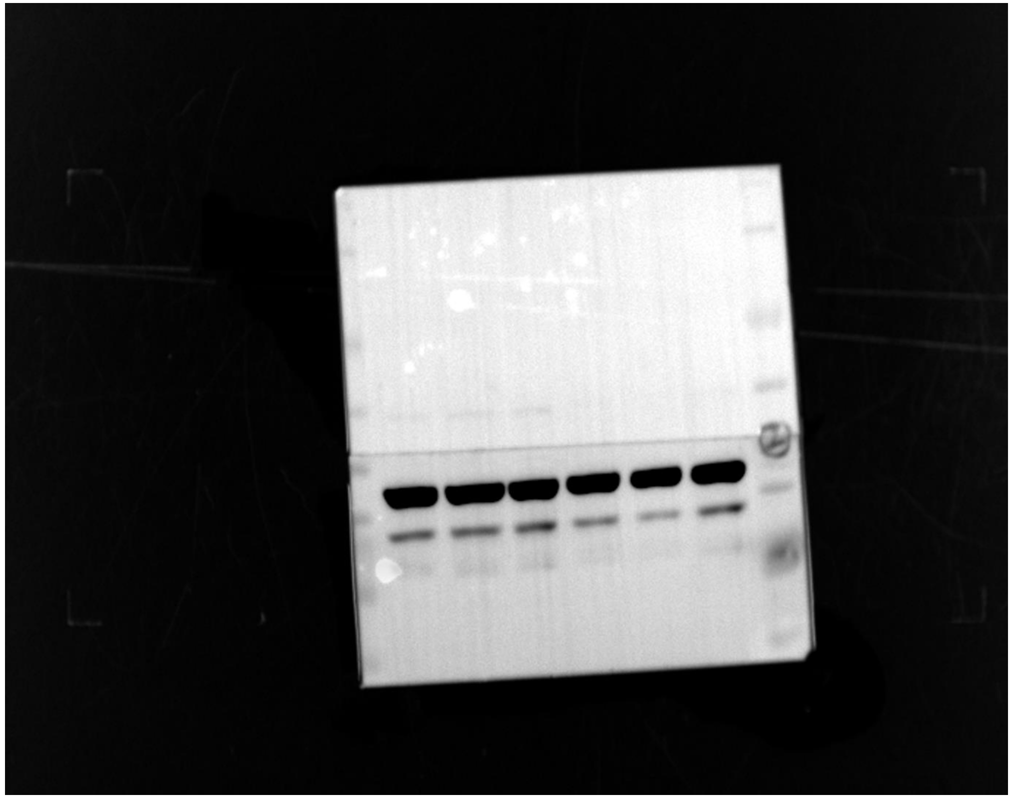

GAPDH

Fig.S4D

NRF2

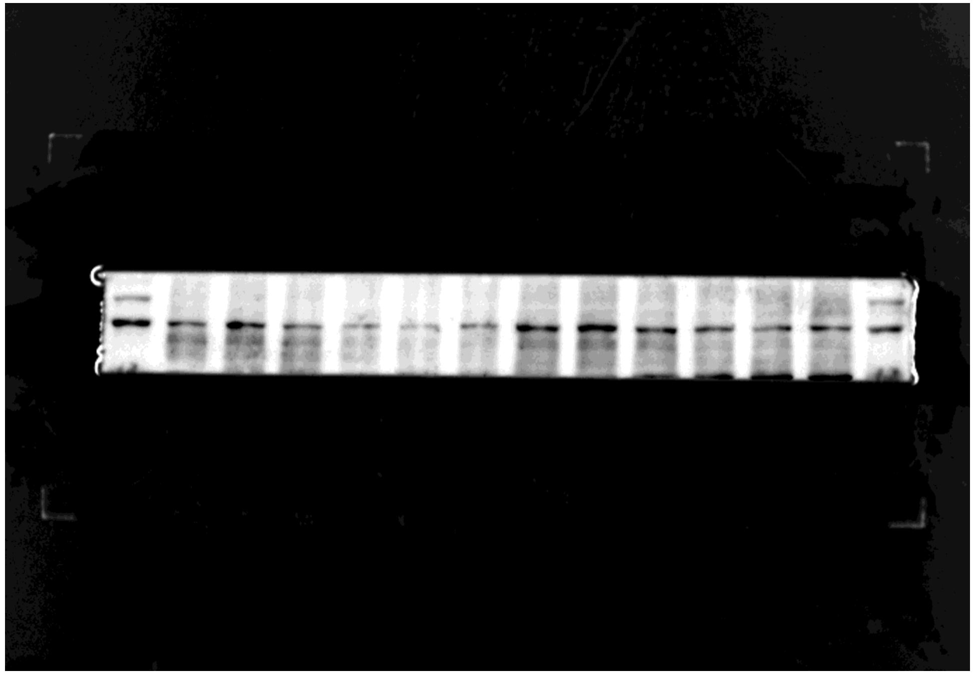

Ugt1a1

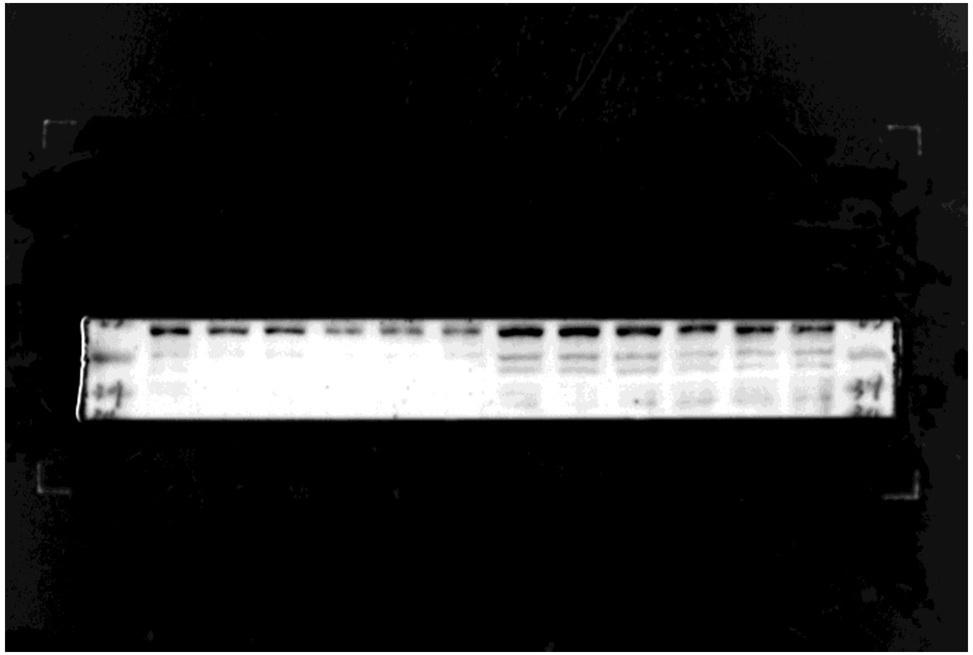

GSTM2

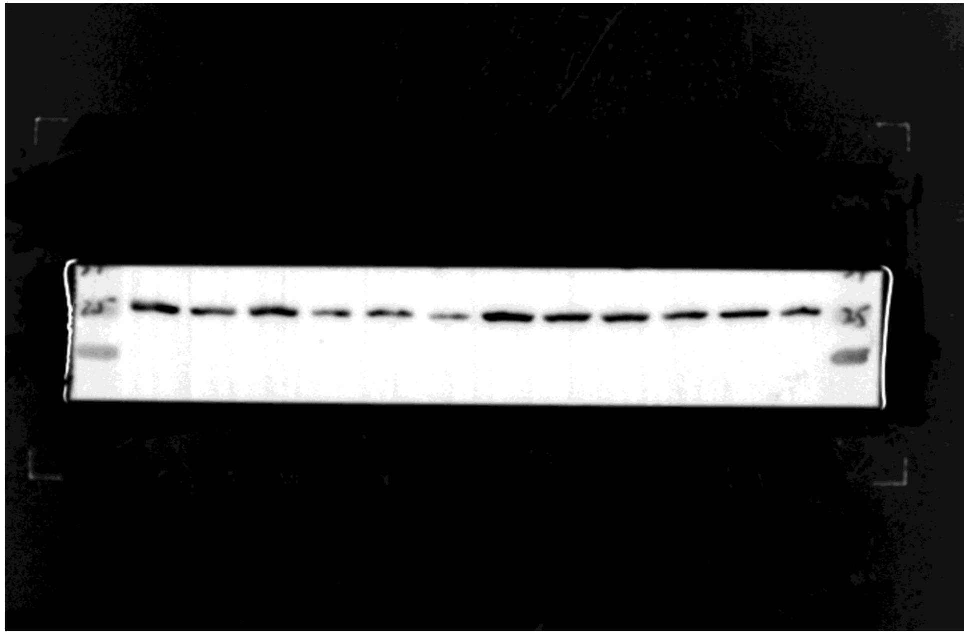

β-actin

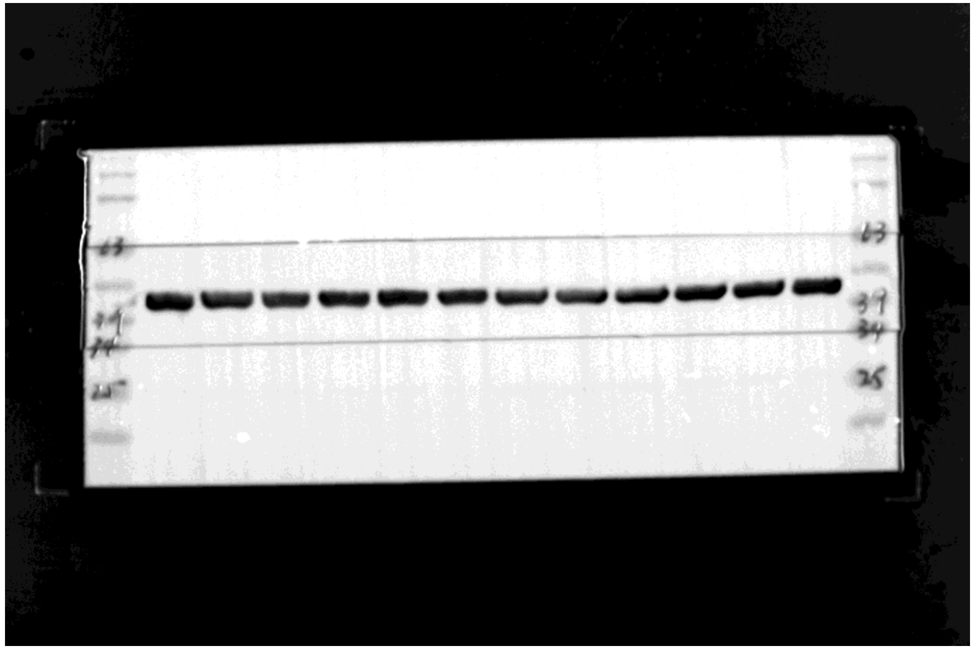

Merge

Fig.4H and Fig.S6D

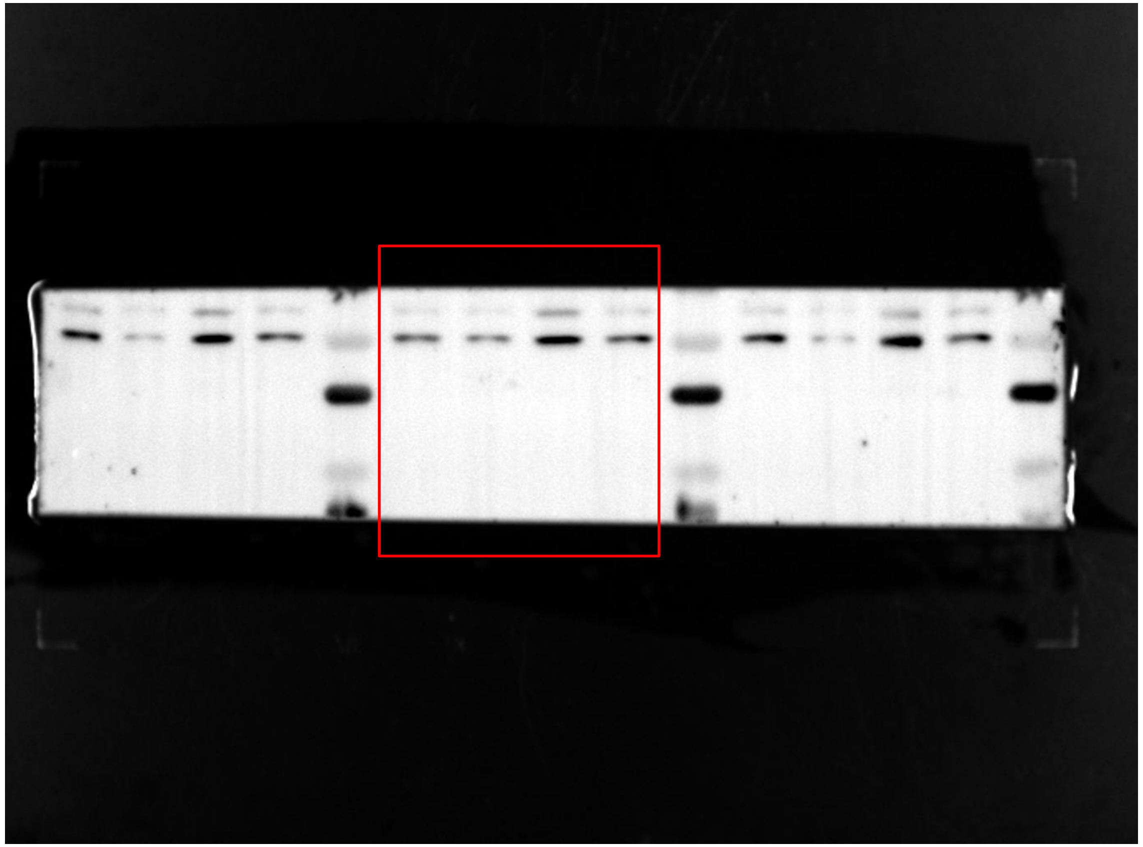

GSTM2

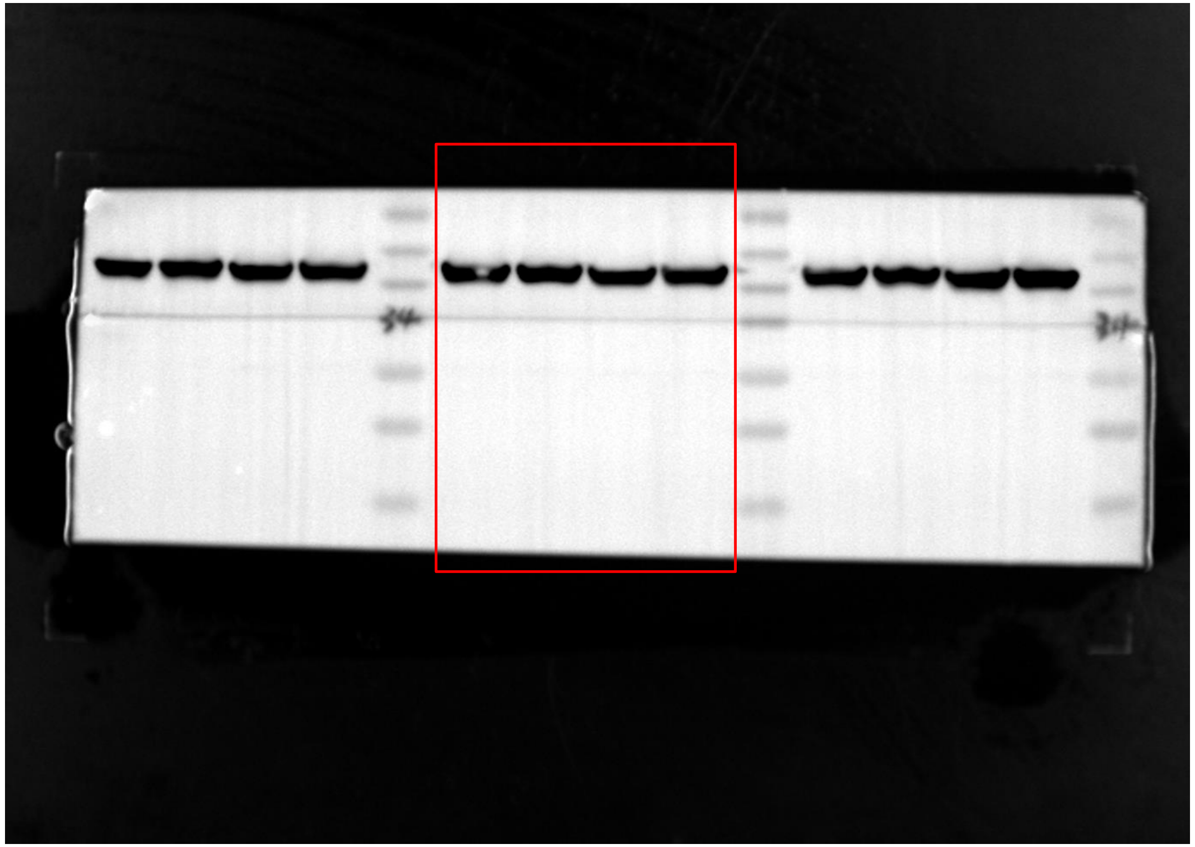

β-actin

Fig.4F

NRF2

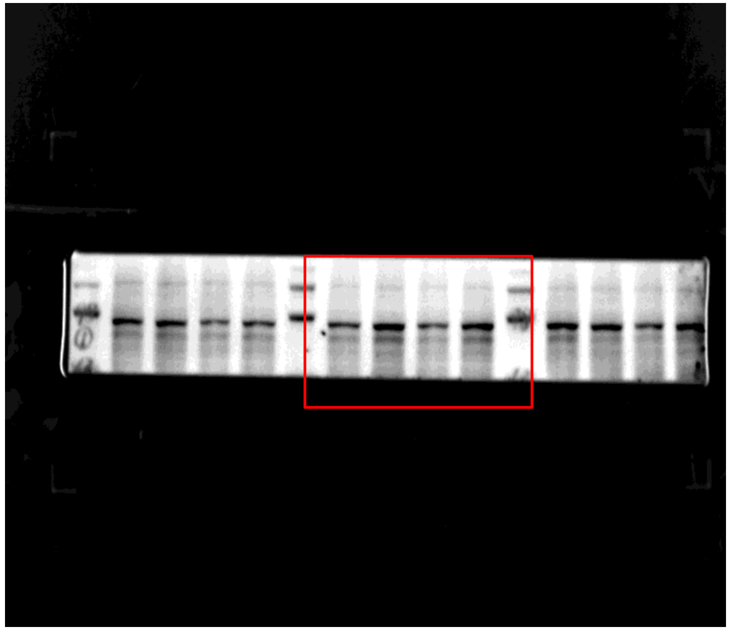

Ugt1a1

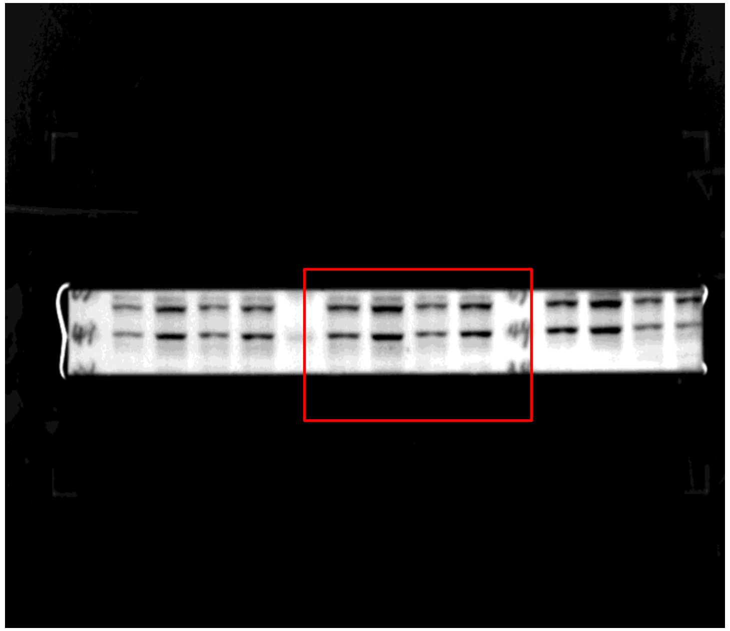

β-actin

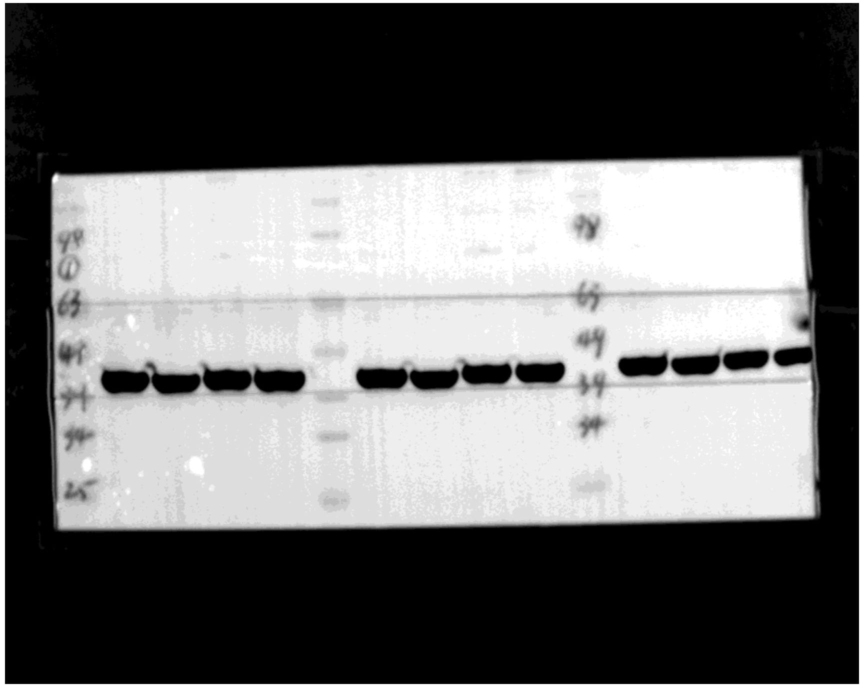

Merge

Fig.4F and Fig.6G

GSTM2

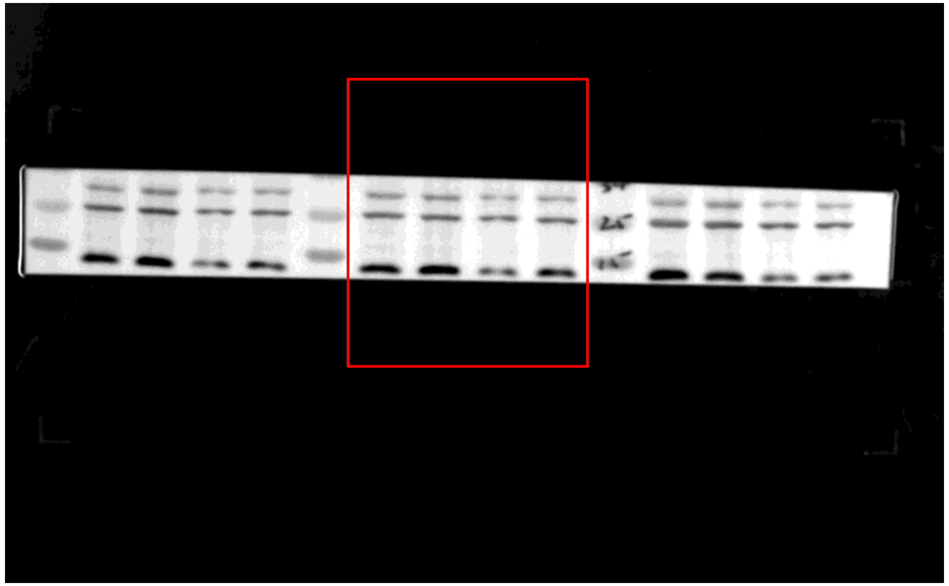

Ugt1a1

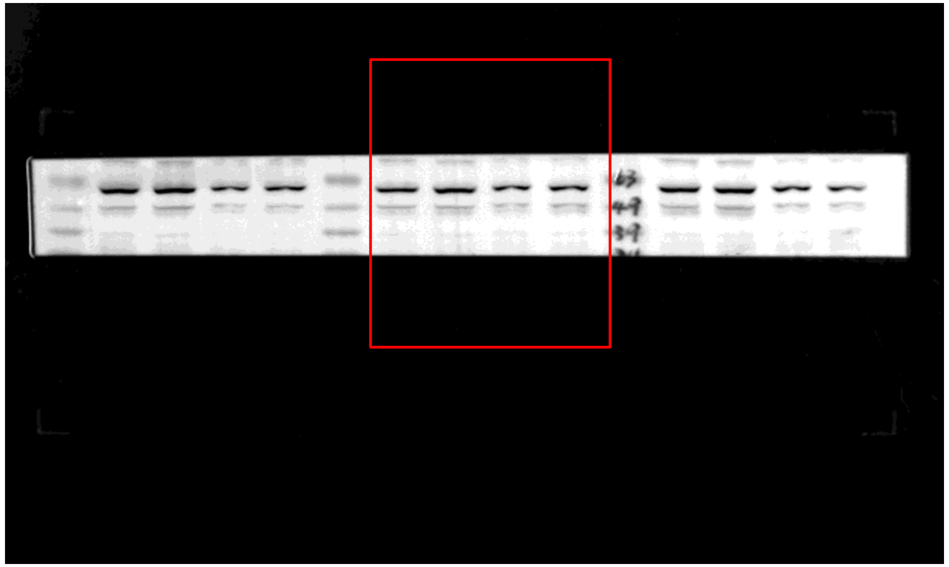

β-actin

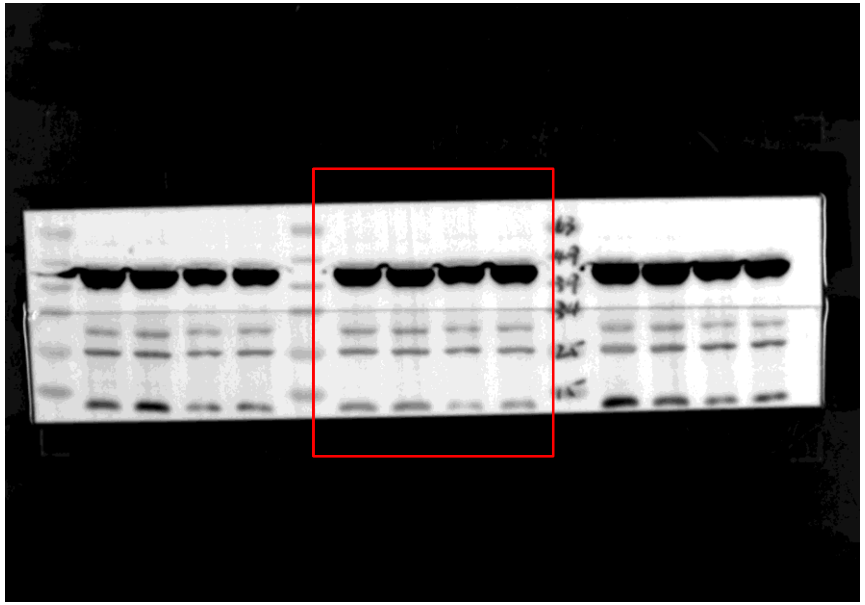

Merge

Fig.4I

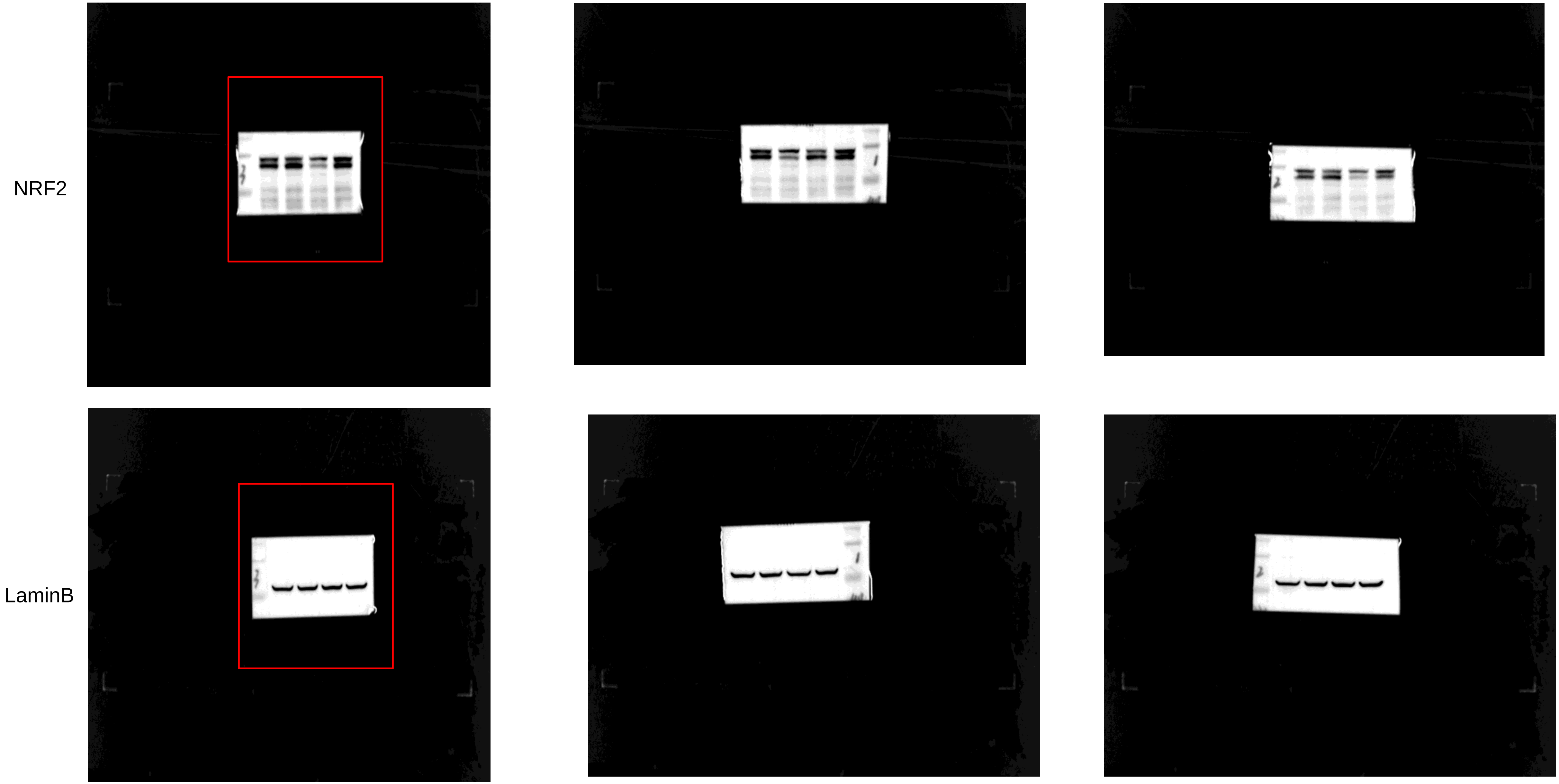

Fig.6G

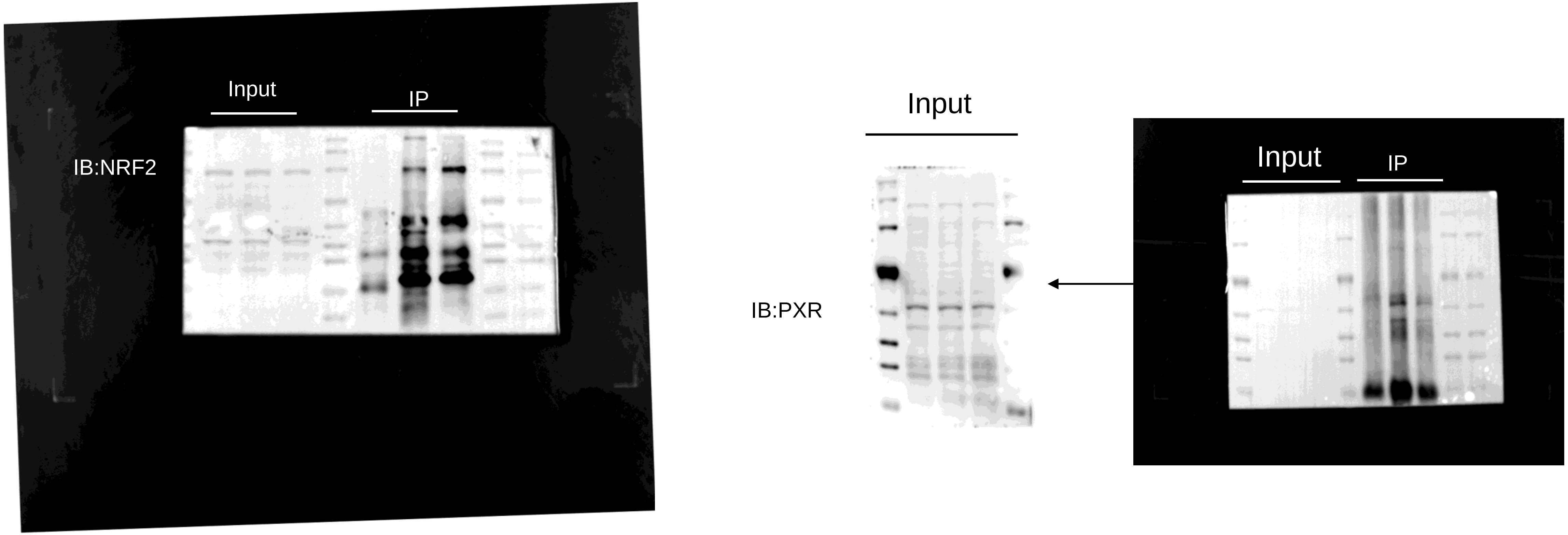

Fig.6J

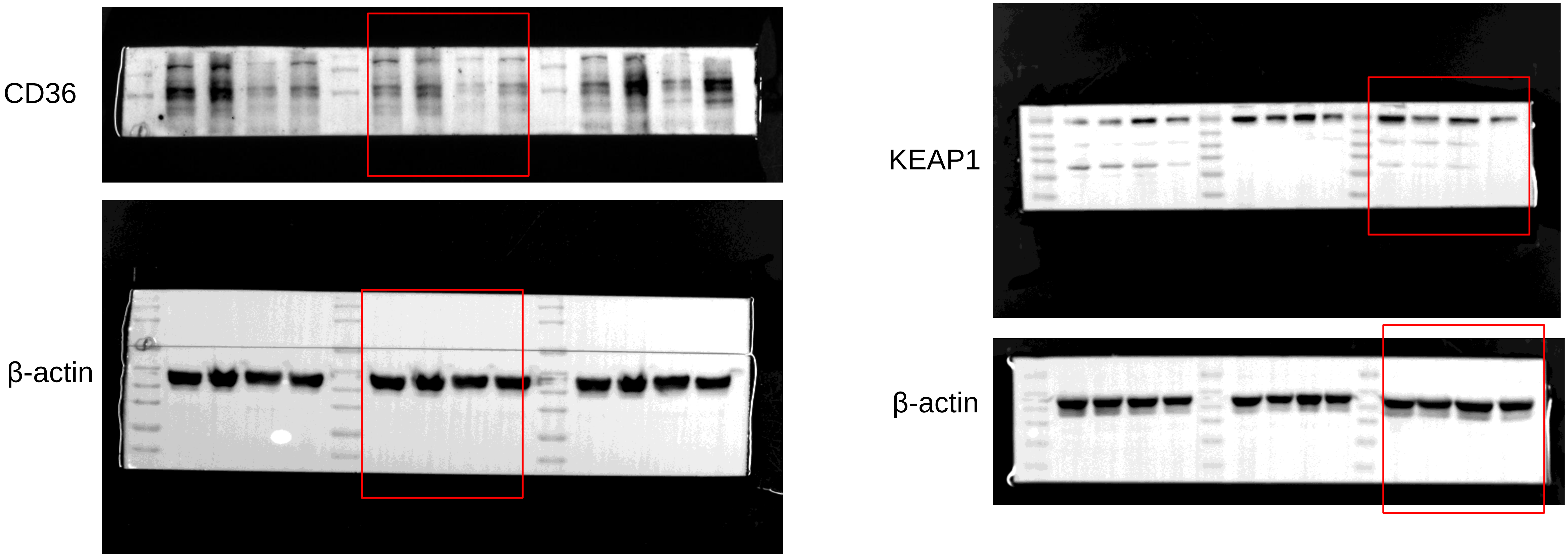

Fig.6G

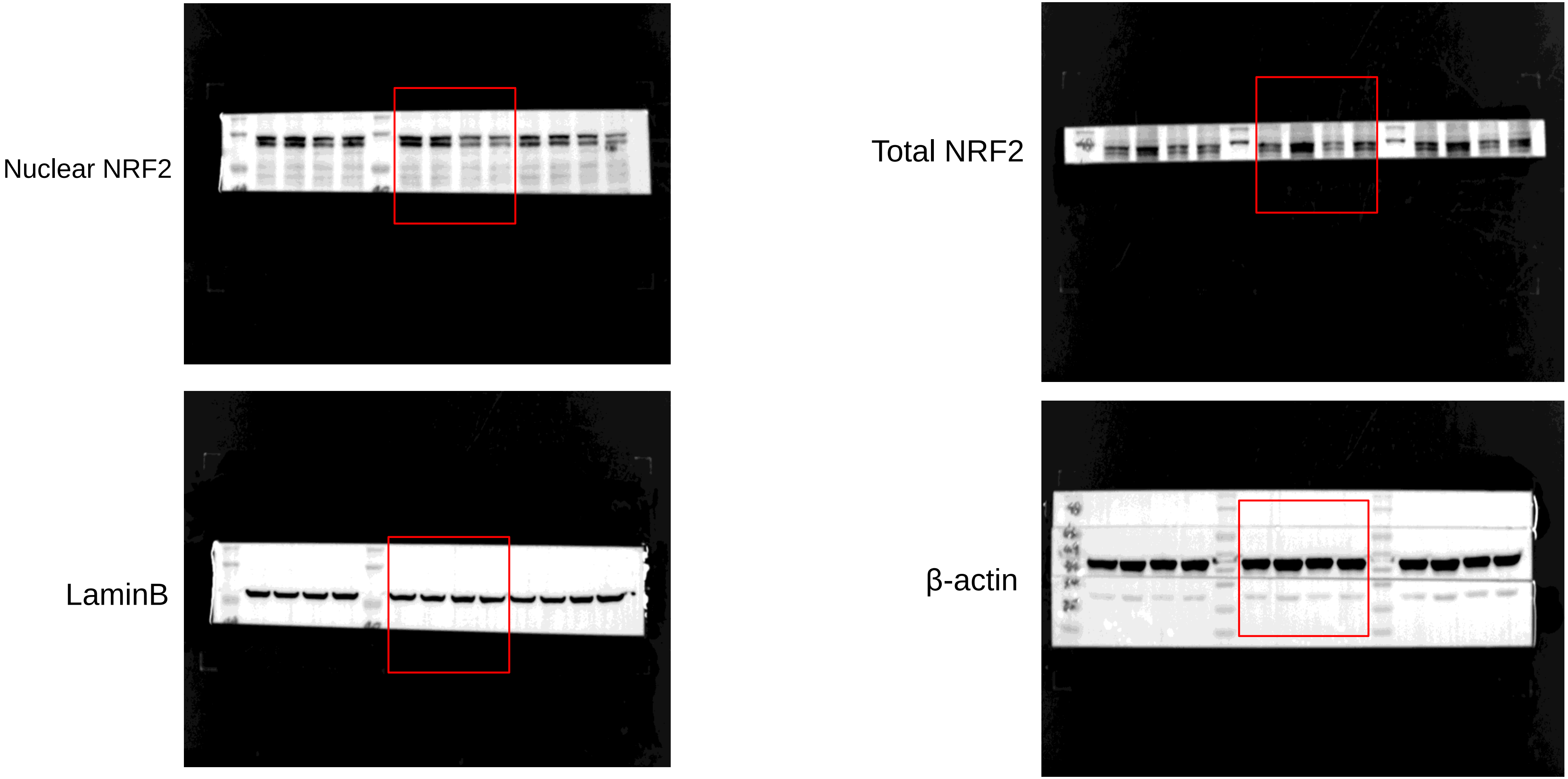

Fig.7C

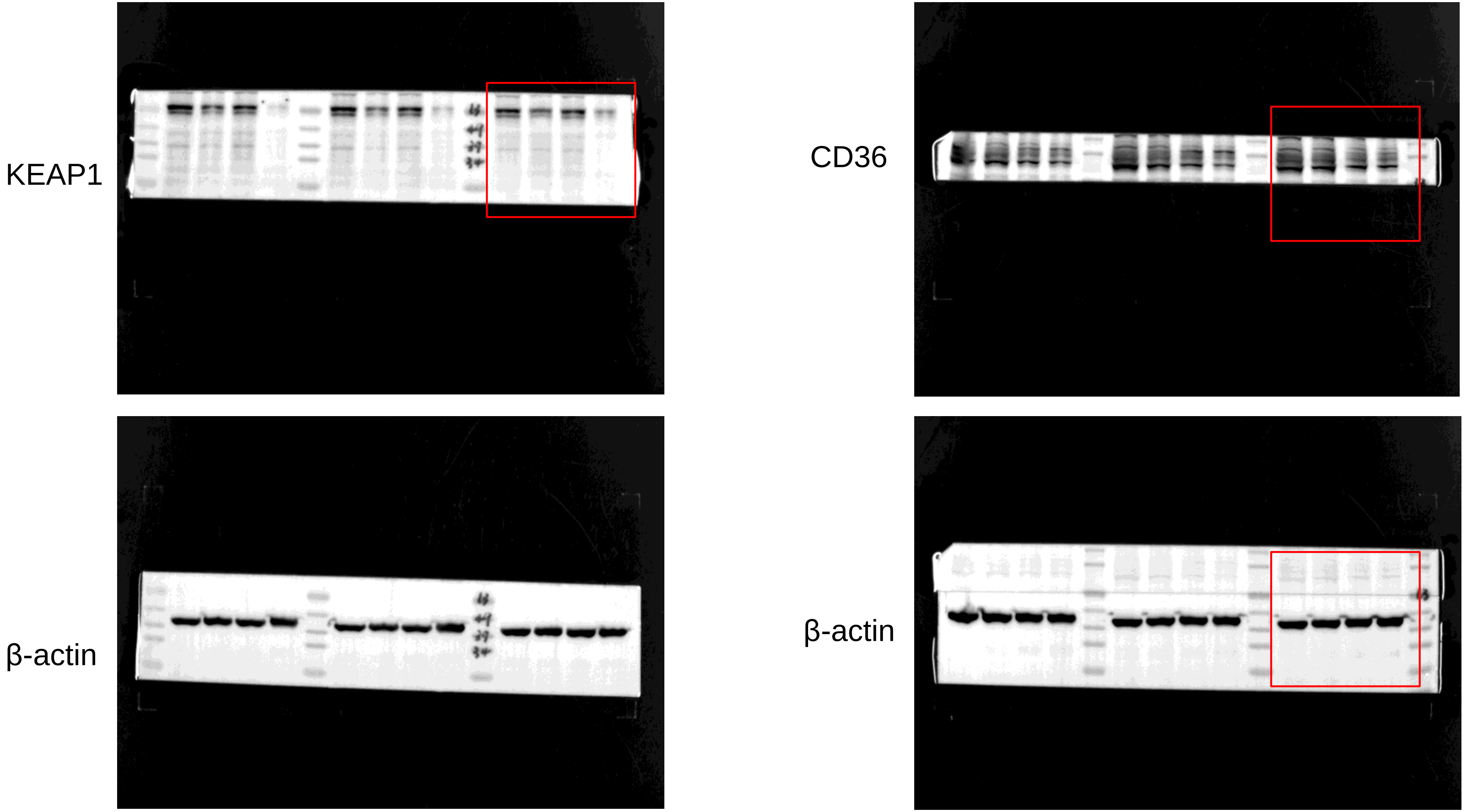

Fig.7C

CD36

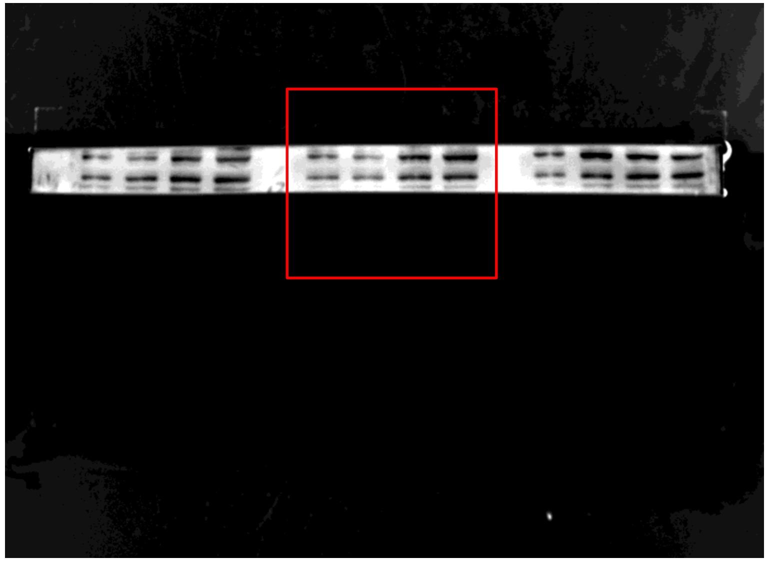

β-actin

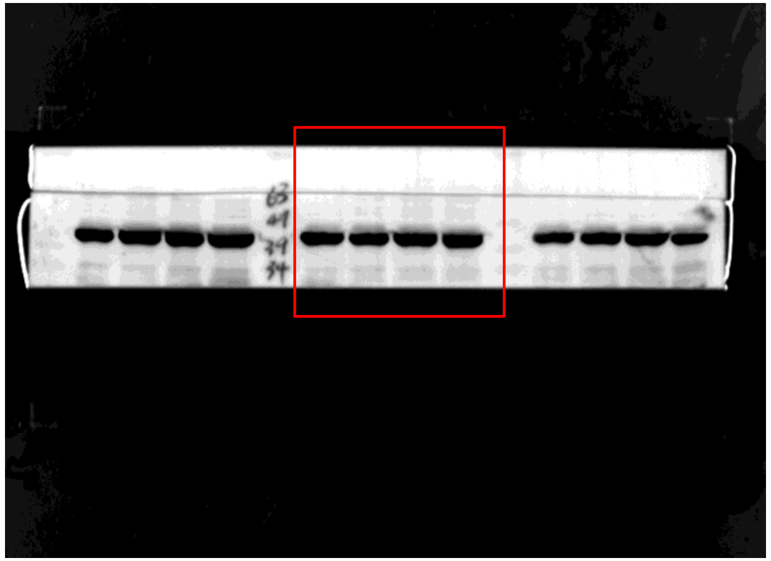

Fig.7C

HO-1

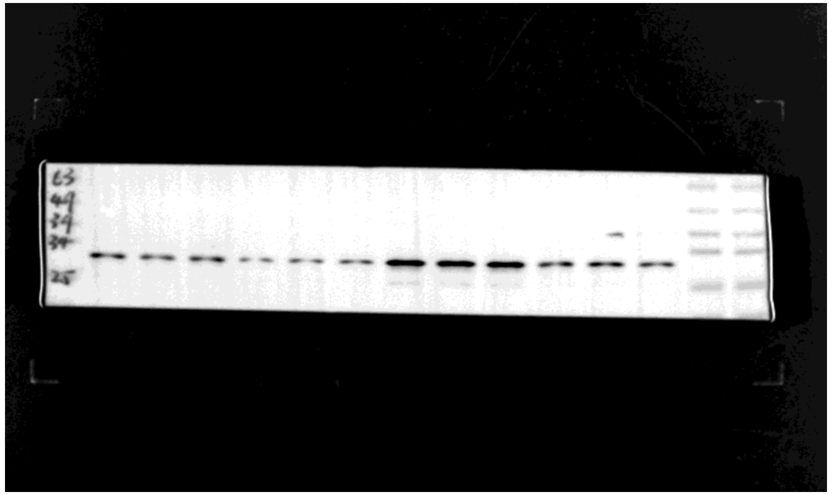

NQO1

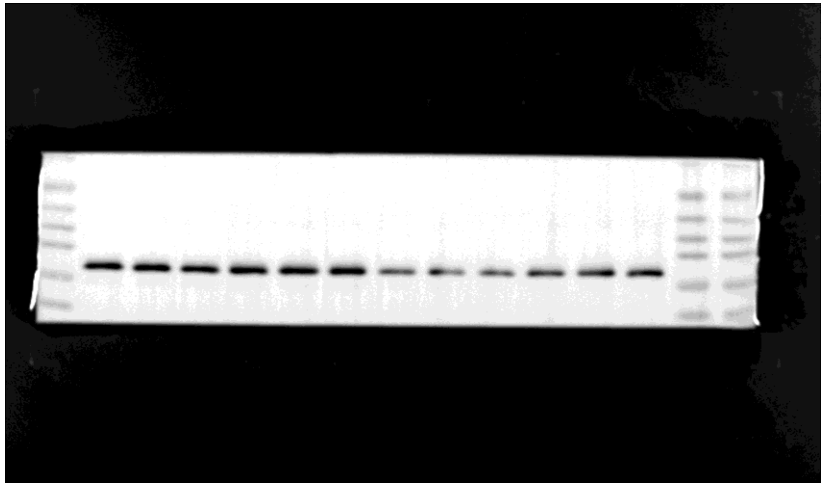

β-actin

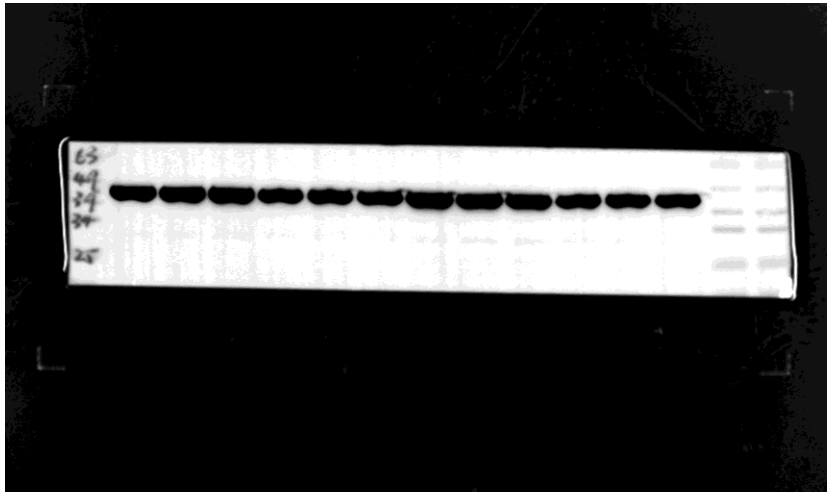

β-actin

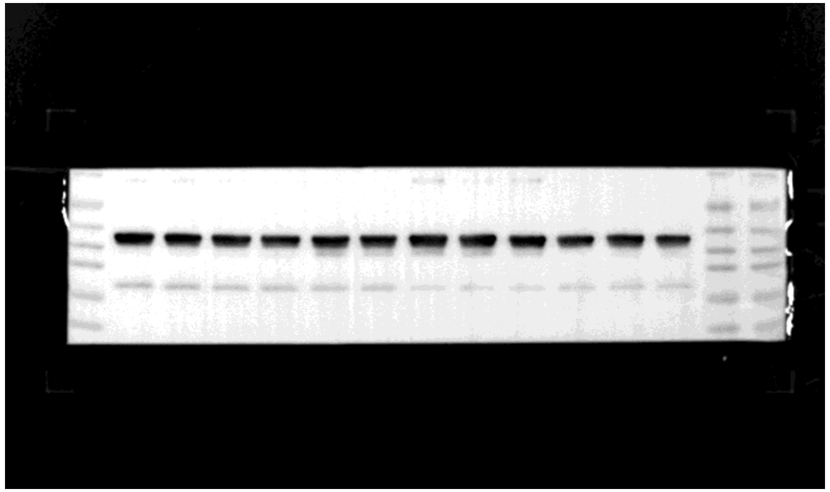

Fig.S6D

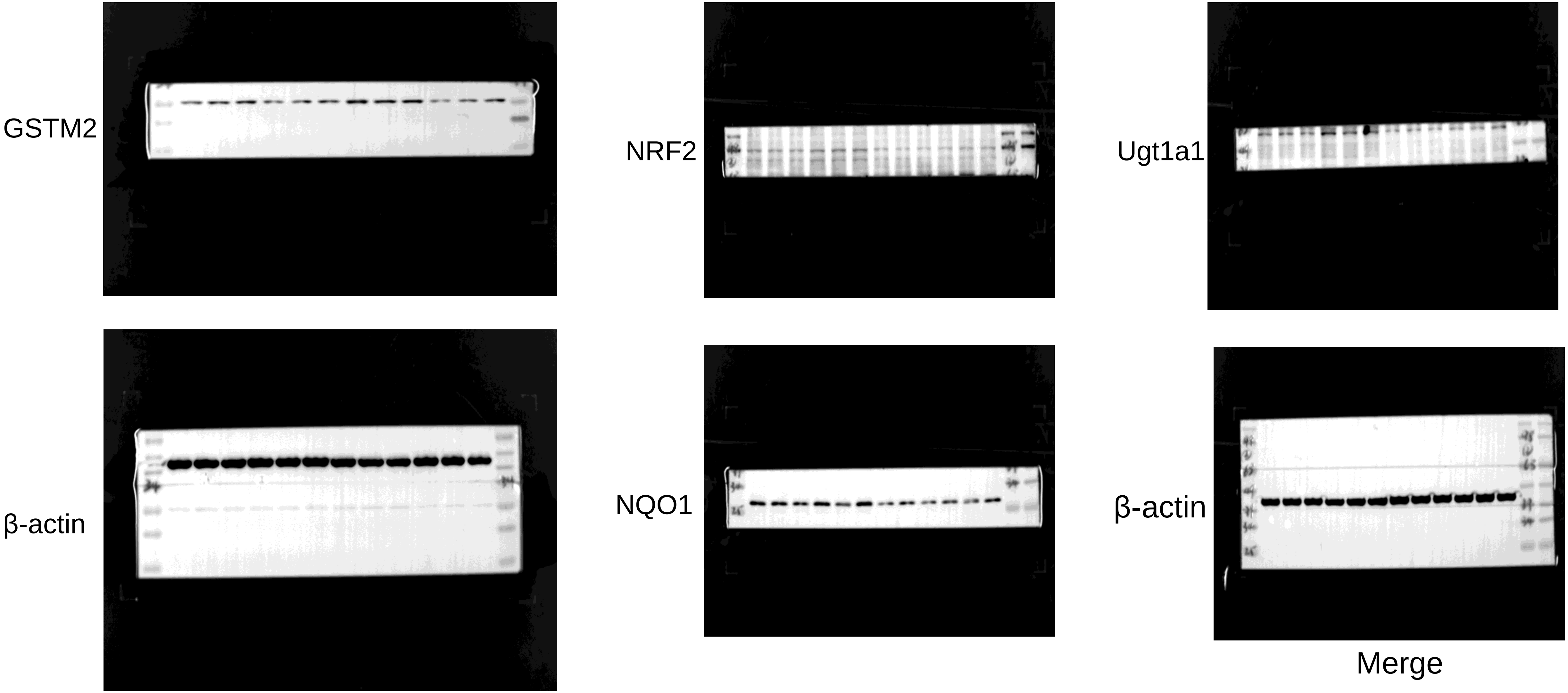

Fig.S6J

HO-1

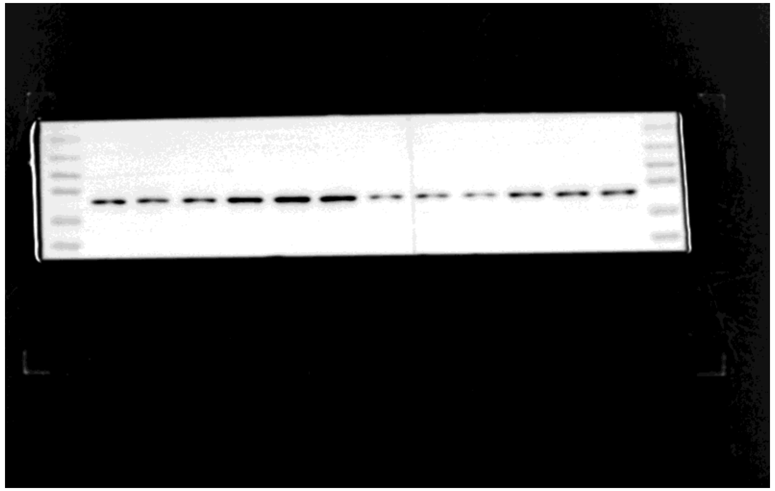

HO-1

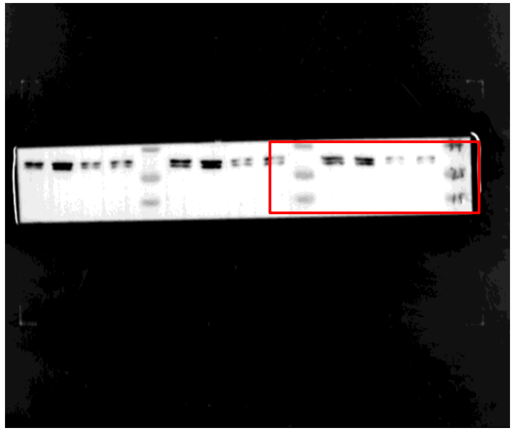

NQO-1

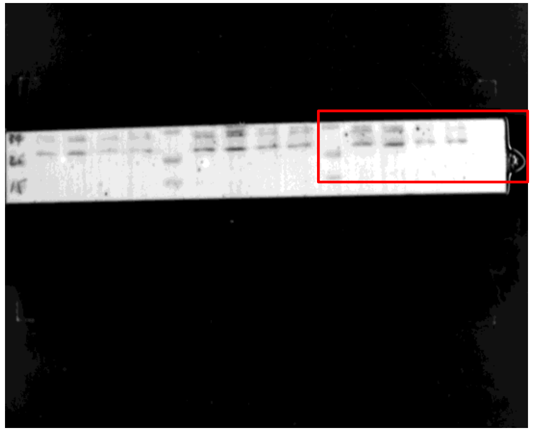

β-actin

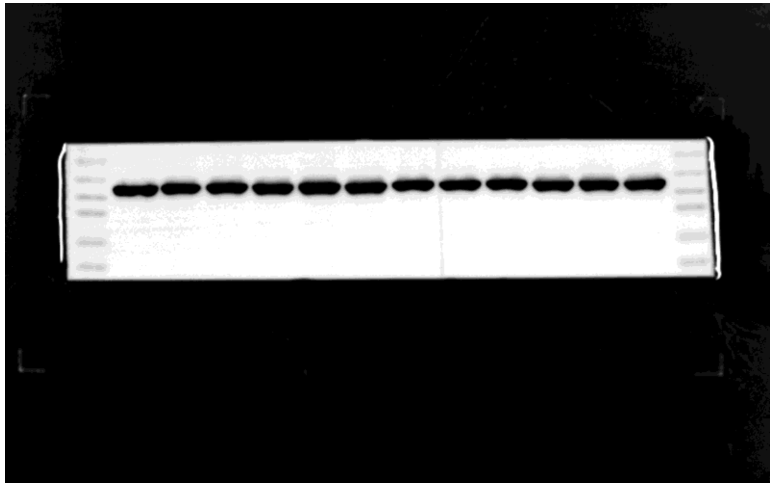

β-actin

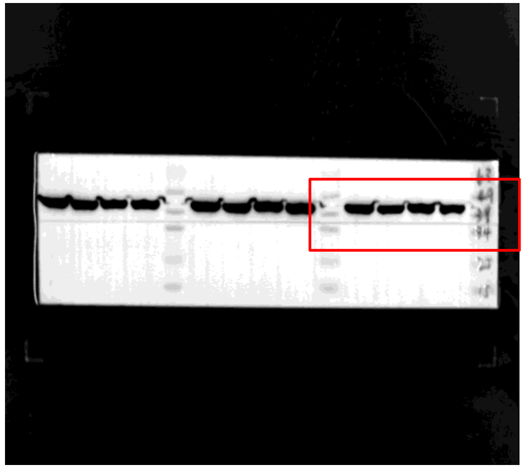

β-actin

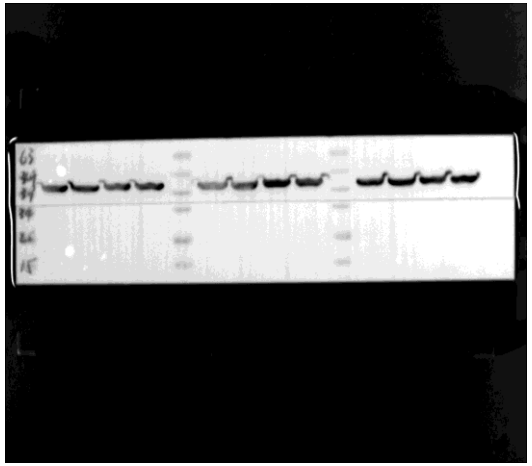

Fig.S6J and S6F

Ugt1a1

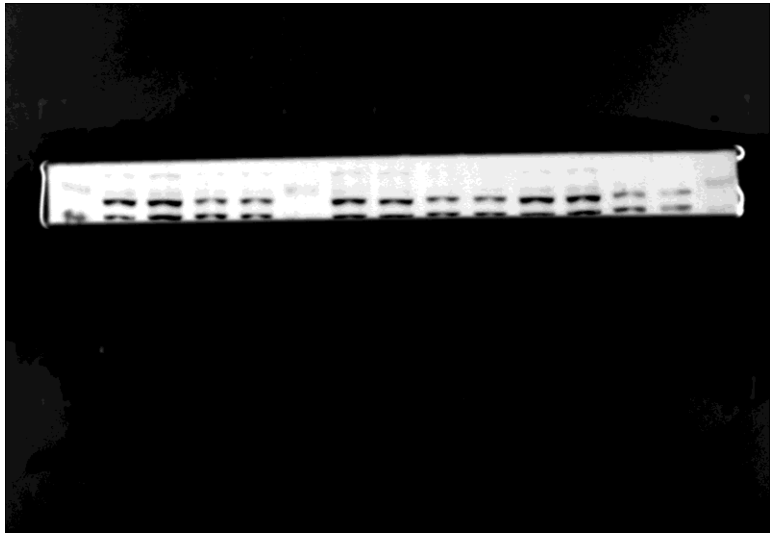

GSTM2

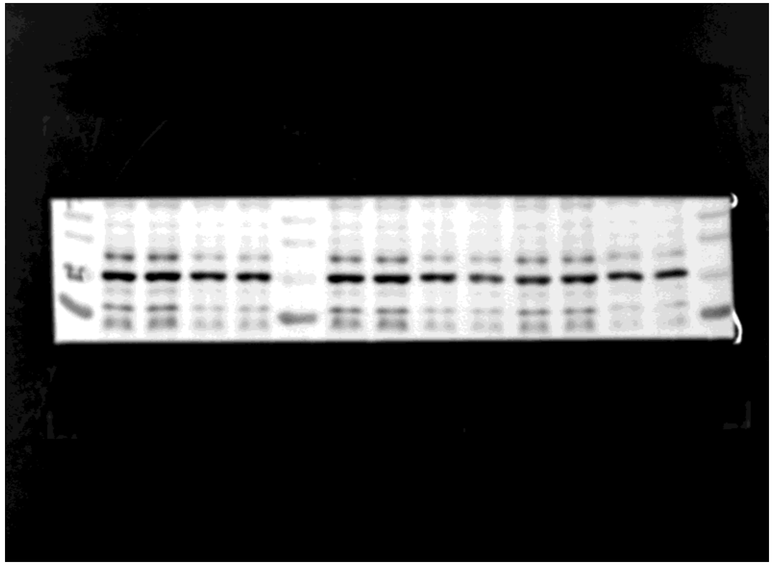

β-actin

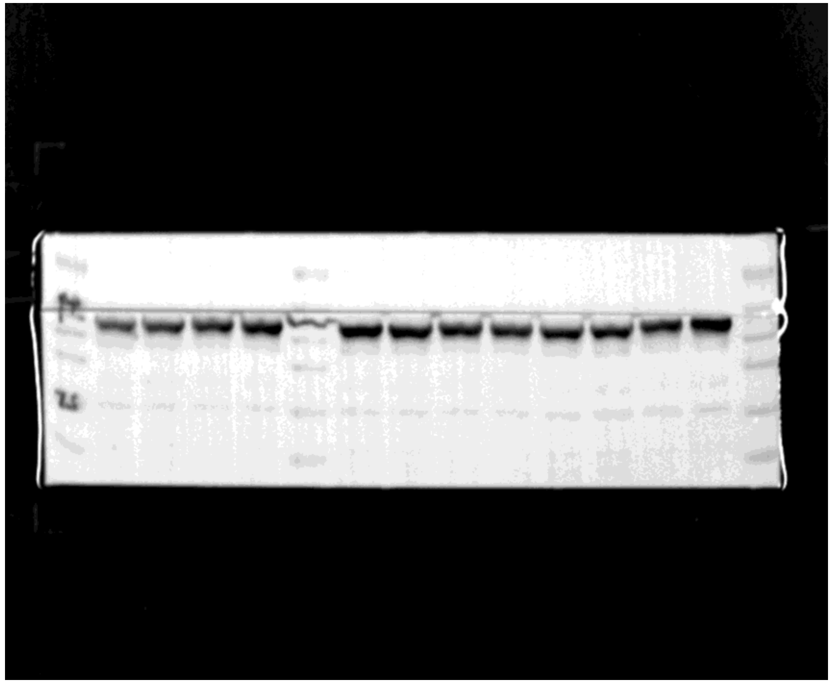

Fig.S7I
