# Supplementary material for: Indole-acetaldehyde from Rothia mucilaginosa activates the PXR/NRF2 axis to enhance alveolar macrophage phagocytosis and protect against ARDS
Source: Respir Res. 2026 Feb 19;27:112. doi: 10.1186/s12931-026-03551-3 (PMC12961788; doi:10.1186/s12931-026-03551-3)
Supplement: Supplementary file 3 — Supplementary Material 3. [file 12931_2026_3551_MOESM3_ESM.docx]

**Supplementary Methods**

1. Cell Culture

MH-S alveolar macrophages, RAW264.7 macrophages, THP-1 monocytes, and A549 lung adenocarcinoma epithelial cells were obtained from Fuheng Biology (Shanghai, China). MH-S, RAW264.7, and THP-1 cells were maintained in RPMI-1640 medium supplemented with 10% fetal bovine serum (FBS); THP-1 cultures additionally contained 0.05 mM β-mercaptoethanol. A549 cells were cultured in DMEM containing 10% FBS. All cell lines were incubated at 37 °C in a humidified atmosphere of 5% CO₂.

2. Bacterial Culture

The standard strain of *R. mucilaginosa* was obtained from BeNa Culture Collection (ATCC 25296; Henan, China). After streaking onto blood agar plates, single colonies were isolated and expanded in Luria-Bertani (LB) broth. The bacterial suspension was diluted proportionally, and optical density was measured at 600 nm (OD600). Colony-forming units (CFUs) were determined by plate counting, and a standard curve correlating OD600 with CFU was generated. Bacterial suspensions were then adjusted to the required concentrations for interventions. In parallel, culture supernatants (10^8^ CFU/mL) and heat-inactivated bacterial preparations were collected for experimental use.

3. Untargeted Metabolomic Profiling Extraction Protocol

Liquid Chromatography-Mass Spectrometry (LC-MS) was used for Untargeted Metabolomic (APTBIO, China). Briefly, samples were thawed at 4 °C, extracted with prechilled methanol/acetonitrile/water (2:2:1, v/v/v), vortexed, and sonicated on ice for 30 min. After incubation at −20 °C for 10 min, mixtures were centrifuged at 14,000 g for 20 min at 4 °C. Supernatants were vacuum-dried, reconstituted in 100 μL acetonitrile/water (1:1, v/v), vortexed, and centrifuged again under the same conditions. The resulting supernatants were used for metabolomic analysis. Chromatographic separation was performed on a HILIC column using Agilent 1290 Infinity or Vanquish UHPLC systems, and mass spectra were acquired on AB Triple TOF 6600 and Q Exactive platforms for comprehensive MS1 and MS2 profiling.

4. Determination of IAAld Concentration

The IAAld reference standard (Yuanye Bio-Technology, China) was dissolved and diluted to 1 mg/mL. Consistent with the observed orange coloration of the standard solution, characteristic absorption peaks of IAAld were identified using UV spectroscopy, with a major absorption maximum at 470 nm. Serial dilutions of IAAld were prepared, and absorbance at 470 nm was measured using a microplate reader to generate a standard concentration–absorbance calibration curve. A linear relationship between IAAld concentration and absorbance was confirmed (R² = 0.9976), and this curve was subsequently used to quantify IAAld concentrations in cell culture supernatants. Additionally, as the unstable chemical nature of IAAld, all solutions are freshly prepared before experiment and stored under 4℃ conditions to minimize their degradation.

5. Flow Cytometry Analysis

BALF samples were processed for flow cytometry as follows: approximately 5 mL of BALF was collected by rinsing with pre-cooled PBS. After cell counting, samples with a total cell number >1×10⁵ were used for further analysis. Cells were centrifuged, resuspended, and incubated with Fc-block (BD Pharming, USA) to block Fc receptors at 4 °C for 10 min. Subsequently, Siglec-F (#155509, BioLegend, USA) , CD11c (#117337, BioLegend, USA) or Ly6G(#127605, BioLegend, USA) was added, followed by incubation at 4 °C in the dark for 30 min. After staining, cells were washed once with flow cytometry buffer and resuspended in 200 μL of fixation solution. Samples were stored protected from light until flow cytometric acquisition.

6. RNA Extraction and qRT-PCR

Total RNA was extracted using a chloroform-free RNA extraction kit (Vazyme, China) according to the manufacturer’s instructions. Briefly, after treatment, cells were washed with PBS, lysed in 1 mL Trizol substitute for 10 min at room temperature, and centrifuged. The supernatant was mixed with 0.2 mL chloroform substitute (1:5, v/v), vortexed, incubated for 10 min, and centrifuged again. The aqueous phase was collected, precipitated with an equal volume of isopropanol, washed with 75% DEPC-treated ethanol, air-dried, and resuspended in nuclease-free water. RNA samples were diluted to 500 ng/μL for cDNA synthesis and subsequent qPCR analysis. The primer sequences used in this study are listed in Table S1.

7. siRNA Transfection

MH-S cells were cultured in antibiotic-free medium until 70–80% confluent. Transfection was carried out using 100 nM siRNA and Lipofectamine 2000 (Thermo Fisher) according to the manufacturer’s protocol. siRNA–lipid complexes were prepared at a 1:1 ratio in serum-free medium, incubated for 20 min at room temperature, and added to cells for 6 h. The medium was then replaced with complete medium, and cells were collected 24–72 h post-transfection for analysis. For intervention experiments, treatments were applied 24 h after transfection.

8. Immunofluorescence (IF) Staining

Lung tissues were fixed in paraformaldehyde, embedded in paraffin, and sectioned. Sections were deparaffinized, rehydrated, and subjected to antigen retrieval in citrate buffer. After blocking with BSA for 1 h, sections were incubated overnight at 4 °C with primary antibodies (sources in Table S2). The next day, sections were washed and incubated with fluorophore-conjugated secondary antibodies for 1 h, followed by DAPI counterstaining. Images were captured using a fluorescence microscope (Leica, Germany).

9. Nuclear Protein Extraction and Western Blotting

Nuclear proteins were extracted using the Nuclear Protein Extraction Kit (Beyotime, China) following the manufacturer’s protocol. Cells were swollen under hypotonic conditions to release cytoplasmic proteins, and nuclei were pelleted by centrifugation. The nuclear pellet was extracted with high-salt reagent after resuspension in lysis reagent A (containing 1 mM PMSF) and vortexing every 3 minutes for 15 minutes on ice. Cytoplasmic proteins were discarded after centrifugation, and nuclear proteins were extracted using nuclear protein extraction reagent, vortexed, incubated on ice, and centrifuged. The nuclear protein-containing supernatant was collected and quantified using a BCA assay. Samples were prepared with protein loading buffer for subsequent Western blot analysis.

For protein extraction from lung tissue or MH-S cells, RIPA buffer (containing 1% PMSF) was used. Protein samples were separated by SDS-PAGE (8-12% acrylamide gels) and transferred to PVDF membranes (0.45 µm). After blocking with 5% non-fat milk for 1 h, membranes were incubated overnight with primary antibodies at 4 °C (listed in Table S2). After washing, membranes were incubated with secondary antibodies for 1 h at room temperature. Chemiluminescent detection was performed using an ECL kit (Epizyme Biotech, China), and images were acquired with a Tanon 5200 automatic imaging system.

10. Co-Immunoprecipitation (Co-IP)

Cells were harvested by centrifugation, resuspended in lysis buffer (Beyotime, China), and incubated on a shaker at 4°C. Protein agarose beads were washed and resuspended at 4°C. After lysing, the samples were centrifuged at 12,000 rpm for 15 minutes at 4°C, and the supernatant was transferred to a new tube. An aliquot of 70 μL was saved for input, and the remaining lysate was incubated overnight with IgG, NRF2, or PXR antibodies and washed beads at 4°C. The next day, the mixture was centrifuged at 2,000 rpm for 5 minutes, and the pellet was washed with non-enzymatic lysis buffer before preparing for Western blot analysis.

11. Chromatin Immunoprecipitation (ChIP)

MH-S cells were treated with 1% formaldehyde at 37°C for 10 minutes to crosslink proteins and DNA, and the reaction was terminated by adding 125 mM glycine. Cells were collected by centrifugation at 2,000 g for 5 minutes, resuspended in IP lysis buffer with protease inhibitors, and lysed on ice for 30 minutes with periodic vortexing. The lysate was sonicated, and 40 μL was saved as Input. The remaining lysate (50 μL) was treated with 5 μL proteinase K and 2 μL 5 M NaCl (final concentration 0.2 M NaCl), followed by crosslink reversal overnight at 55°C. After reversing crosslinking, nucleic acid concentration was measured, and a portion was used for PCR and agarose gel electrophoresis to assess sonication efficiency and confirm target DNA presence.

After confirming results, the remaining sample was diluted with 1 mM PMS ChIP buffer and 20 μL of 50× PIC, followed by addition of 60 μL Protein A+G Agarose. The mixture was incubated with rotation at 4°C for 1 hour, then NRF2 or mouse IgG antibodies were added and incubated overnight at 4°C. After washing, DNA was recovered for ChIP-qPCR analysis. Binding sites were predicted using the JASPAR database, and primers were synthesized based on these sites (primer sequences provided in Table S1).

12. Transcriptome Analysis

Transcriptome analysis was performed by LC-Bio Technologies (China). Total RNA was extracted using TRIzol reagent, and its integrity was assessed with a Bioanalyzer 2100 (Agilent, CA, USA). RNA samples with a concentration >50 ng/μL, RIN >7.0, and total RNA >1 μg were used for subsequent steps. PolyA-containing mRNA was isolated using oligo magnetic beads (Dynabeads Oligo, Thermo Fisher) through two rounds of purification. mRNA was then fragmented, and cDNA was synthesized using SuperScript II Reverse Transcriptase and double-stranded cDNA was synthesized with E. coli DNA polymerase I. The resulting DNA-RNA complexes were converted to double-stranded DNA, and the library was prepared with a fragment size of 300 bp ± 50 bp (strand-specific). Paired-end sequencing (PE150) was performed on an Illumina NovaseqTM 6000 (LC-Bio Technologie, China) following standard protocols.

13. Fluorescence In Situ Hybridization (FISH)

Lung tissue from mice was collected, cleaned, and fixed in fixation buffer (DEPC-treated water) for 12 hours. The tissue was then dehydrated, paraffin-embedded, and sectioned. Sections were heated in retrieval buffer for 15 minutes and cooled. Proteinase K (20 μg/mL) was applied and incubated at 37°C for 20-30 minutes, followed by washing. Pre-hybridization solution was added and incubated at 37°C for 1 hour. After removing the pre-hybridization solution, hybridization solution containing the probe was applied, and sections were incubated overnight at 37°C. The following day, sections were washed and counterstained with DAPI. Fluorescent images were captured using a Nikon fluorescence microscope (DAPI: excitation 420 nm, emission blue; CY3: excitation 510-560 nm, emission 590 nm).

14. Molecular Docking

Molecular docking of IAD with PXR was performed using Autodock Vina 1.2.2. The molecular structure of IAD was obtained from the PubChem database (https://pubchem.ncbi.nlm.nih.gov), and the 3D coordinates of mPXR/hPXR (PDB: 9BEQ and 2O9I) were retrieved from the Protein Data Bank (http://www.rcsb.org/). Protein and ligand files were converted to PDBQT format, water molecules were removed, and polar hydrogen atoms were added. A grid box was centered to encompass the protein domains and allow free movement of the molecules. The docking interface pocket was defined as a 30 Å × 30 Å × 30 Å cubic region with a 0.05 nm grid spacing. Docking simulations were conducted using Autodock Vina 1.2.2 (http://autodock.scripps.edu), and the resulting models were visualized for further analysis.

15. DHE Staining

Fresh lung tissue was sectioned and immediately stained with an appropriate volume of DHE staining solution (Beyotime, China). The sections were incubated at 37°C and protected from light for 20 minutes. After incubation, the sections were washed once and observed under a fluorescence microscope. DHE fluorescence was detected in the red spectrum with excitation/emission at 535/610 nm.

16. Multiplex Immunoassay (cytokines detection)

Multiplex immunoassay analysis was performed by ABclonal (China). In brief, antibodies specific to target analytes were covalently coupled to uniquely encoded microspheres, each representing a distinct detection item. Mixed microspheres were incubated with samples, allowing target binding, followed by reaction with fluorophore-labeled detection antibodies. Fluorescence intensity was measured to quantify analyte concentrations. For each assay, 5 μL of microsphere suspension and 50 μL of standard or sample were added to each well and incubated at 37°C. After washing, detection antibody solution and fluorophore were sequentially added, followed by incubation in the dark at 37°C for 15 minutes. Fluorescence was detected using the ABplex-100 system (ABclonal, China).

Table S1. **Sequences for primers and siRNA used in this study.**

| **Primers** | Sequence |
| --- | --- |
| Mus musculus |  |
| NRF2 forward | ACCTCTGCTGCAAGTAGCCT |
| NRF2 reverse | TGGGCAACCATCACTCTGCT |
| CYP3a11 forward | GTGCTCCTAGCAATCAGCTT |
| CYP3a11 reverse | GTCGAATTTCCATAAACCCTTGTA |
| GSTM2 forward | CGAAAGCACAACCTGTGTGGA |
| GSTM2 reverse | CCCTCTAAGTACTCTGGCTTCTTT |
| Ugt1a1 forward | GTGAAAGATTACCCCAGGCCCA |
| Ugt1a1 reverse | ACATAGGCTTCAAATTCCTGGGATA |
| Actin forward | CACTGTCGAGTCGCGTCC |
| Actin reverse | TCATCCATGGCGAACTGGTG |
| Homo sapiens |  |
| CYP3A4 forward H | TGTAAAGAAACACAGATCCCCCT |
| CYP3A4 reverse H | TCCACTTACGGTGCCATCC |
| UGT1A1 forward H | CCAAAATCCACTATCCCAGGAATTT |
| UGT1A1 reverse H | TTTTGCCCAAAGCATCAGCA |
| GSTM2 forward H | GAGAAACCAAGTATTTGAGCCCAG |
| GSTM2 reverse H | AGATCTTCTCCAAGCCCTCAAA |
| Actin forward H | ACAGAGCCTCGCCTTTGC |
| Actin reverse H | GATATCATCATCCATGGTGAGCTGG |
| Others |  |
| si*Pxr* | GCGTCATCAACTTCGCCAA |
| CHIP (site2) forward | TATCCCCCTTGCCTGTCTCA |
| CHIP (site2) reverse | CAGCTTTGGAACAGCACGAC |
| CHIP (site1) forward | TGCTGAGTAAGACCATGGCA |
| CHIP (site1) reverse | TTCAGACAGAGCCTCCCCAC |

Table S2. Primary antibodies **used in this study.**

| **Antibodies** |  |
| --- | --- |
| Pxr | WB 1:5000, IHC 1:100, IF: 1:100, Proteintech, 67912-1-lg, China |
| NRF2 | WB 1:1500, IF 1:100, Abclonal, A3577, China |
| KEAP1 | WB 1:1000, Abclonal, A25951, China |
| GSTM2 | WB 1:1500, Abcam, ab196503, UK |
| Ugt1a1 | WB 1:2000, Abclonal, A22609, China |
| HMOX1 | WB 1:2000, Abclonal, A19062, China |
| NQO1 | WB 1:2000, Abclonal, A22290, China |
| CD36 | WB 1:2000, IF: 1: 150, CST, #28109, USA |
| Lamin B1 | WB 1:1000, Abclonal, A11495, China |
| Siglec F (CD170) | IF 1:100, Invitrogen, #14-1702-80, USA |
| CHRM5 | IHC 1:50, OriGene, TA352006S, USA |
| β-actin | WB 1:10000, Abclonal, AC028, China |
| GAPDH | WB 1:5000, Abclonal, A19056, China |


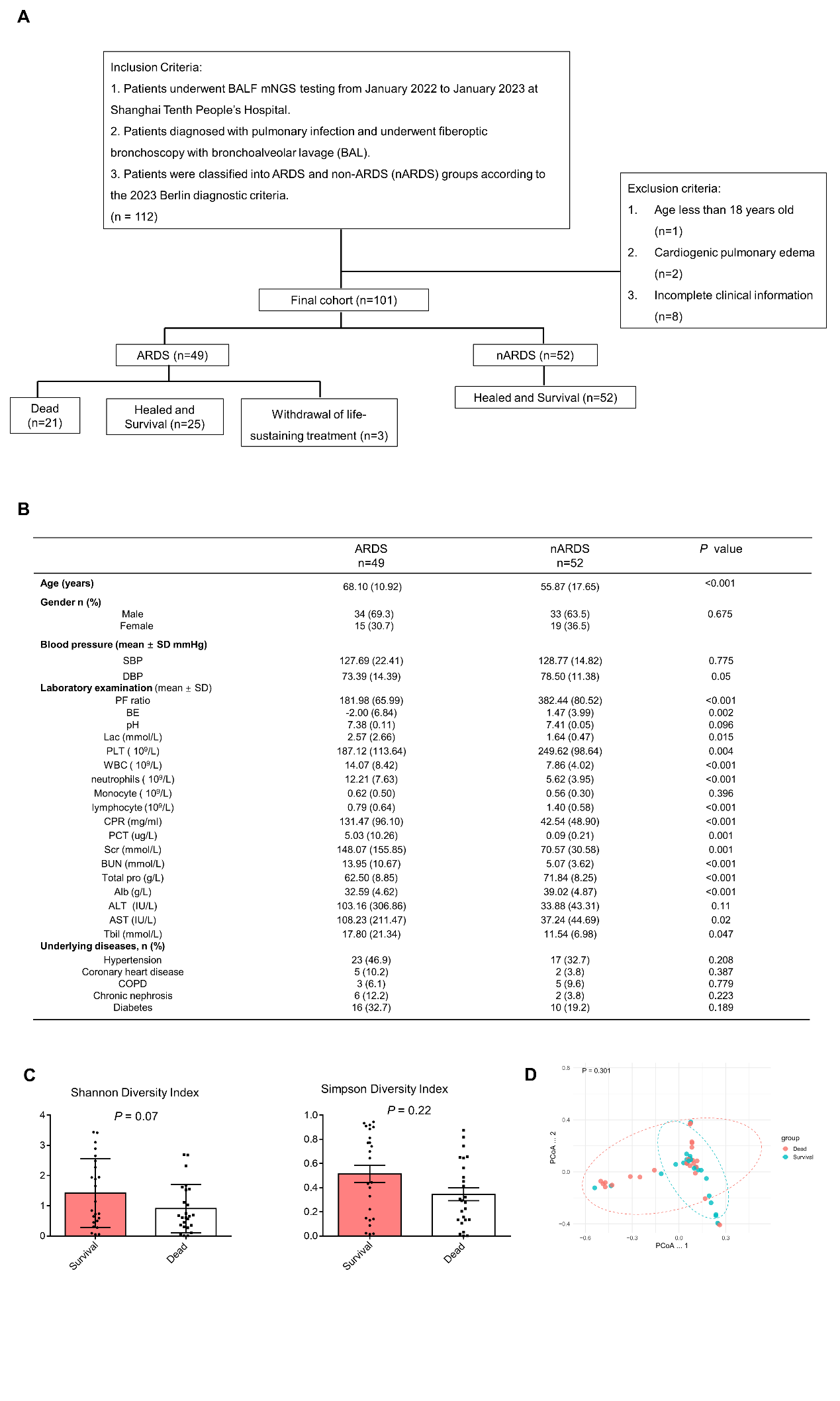


Figure S1. Patient inclusion and clinical baseline characteristics in ARDS and non-ARDS groups. (A) Patient inclusion and exclusion criteria. (B) Clinical baseline characteristics of ARDS (n = 49) and non-ARDS (n = 52) groups. (C) Comparison of α-diversity of the BALF microbiome between ARDS survivors and non-survivors, represented by Shannon index and Simpson index. (D) β-diversity comparison between ARDS survivors and non-survivors, visualized through principal coordinate analysis (PCoA). Data are presented as mean ± SEM.


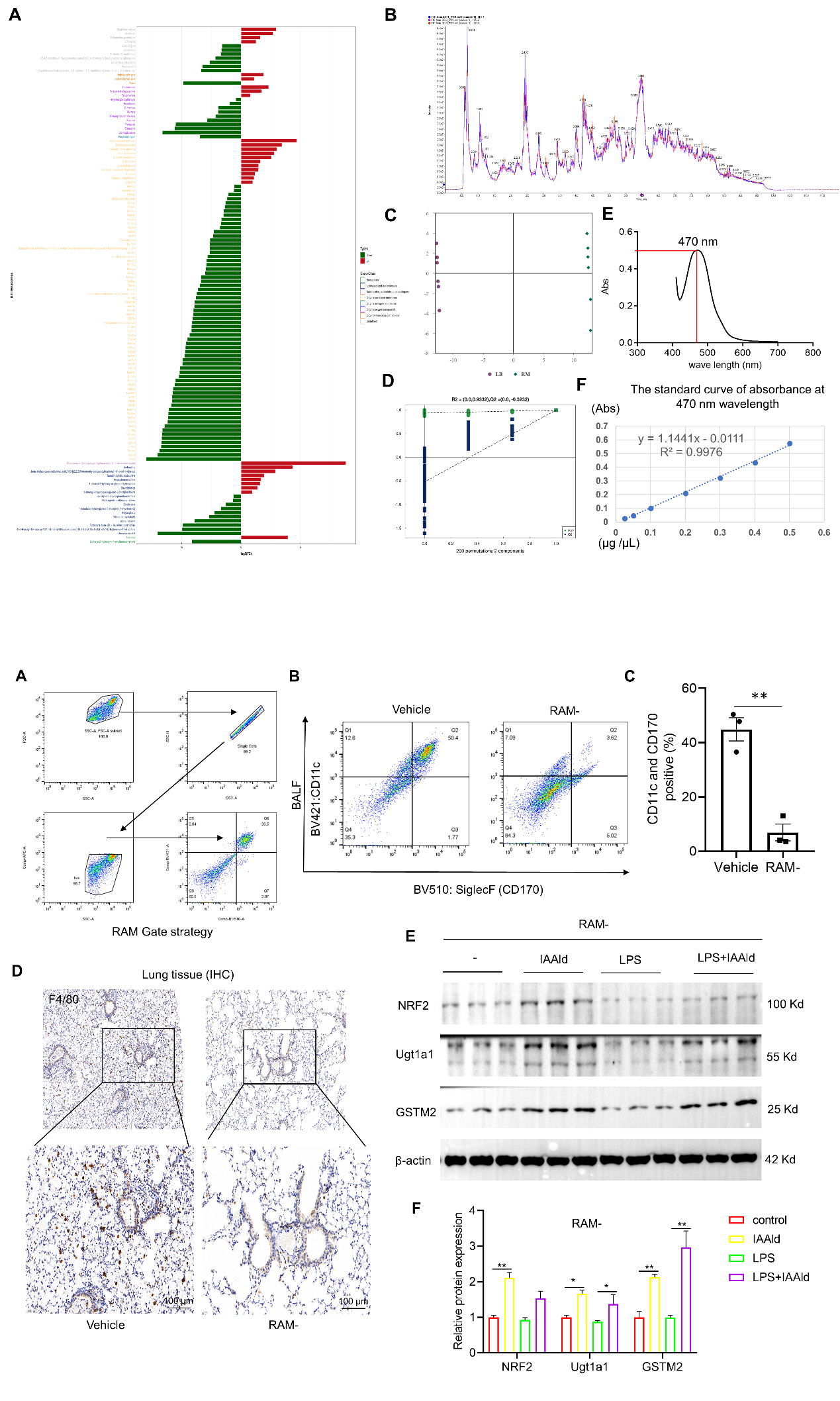


Figure S2. Metabolomic analysis and characterization of IAAld. (A) Differential abundance scores (DA score) of non-targeted cationic and anionic metabolomics analysis, presented collectively. (B) Specific mass spectrometric absorption peaks from non-targeted metabolomics analysis. (C) and (D) Quality control results from PCoA analysis of single metabolite products between two groups. (E) UV-specific absorption peak of IAAld at 470 nm. (F) Absorbance measurements of IAAld at varying concentrations at 470 nm, obtained using a microplate reader to establish a standard curve. Data are presented as mean ± SEM.


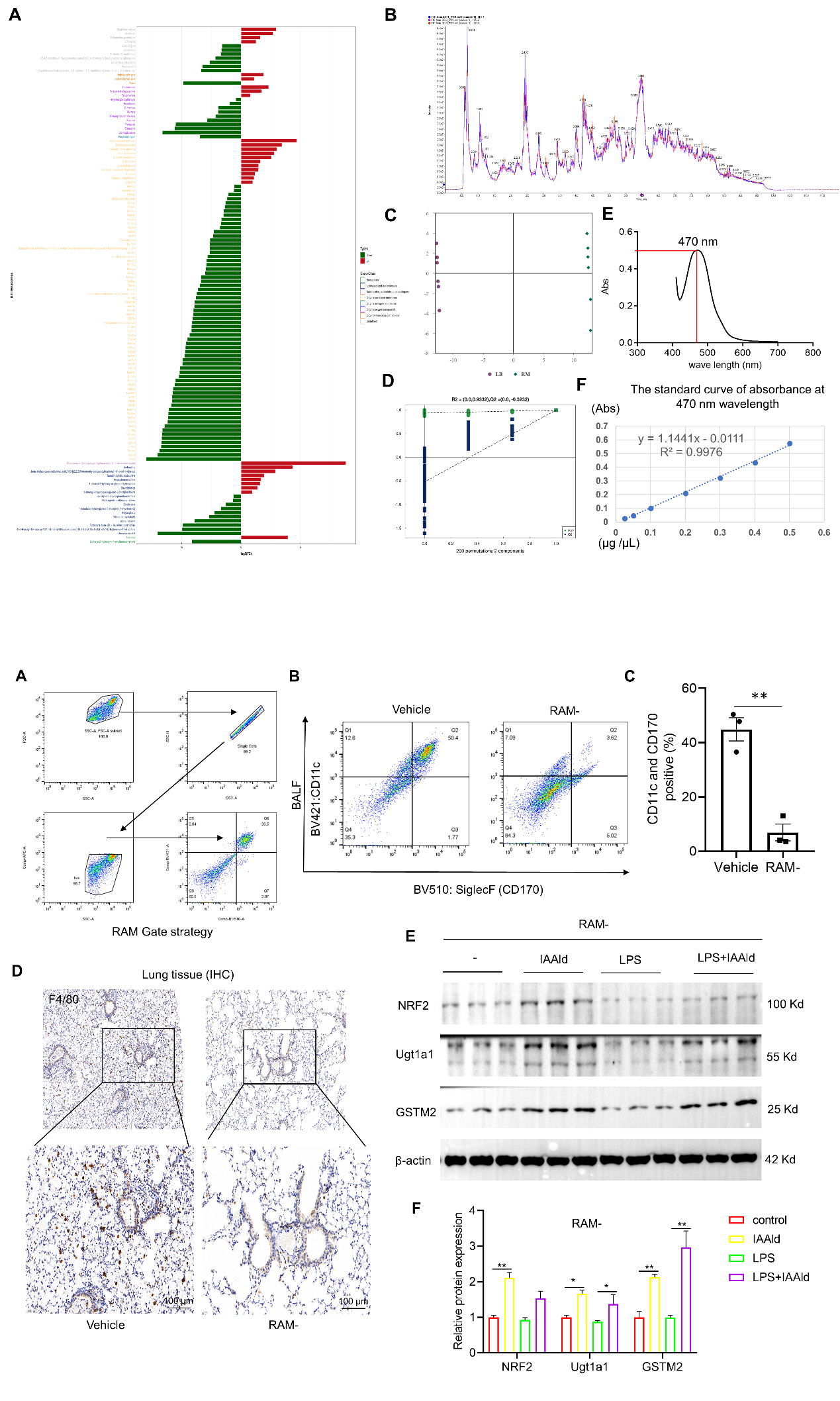


Figure S3. Analysis of RAM clearance and PXR pathway changes after macrophage depletion. (A) Flow cytometry gating strategy for resident alveolar macrophages (RAMs), defined as CD11c and Siglec-F (CD170) double-positive cells. (B) Flow cytometry analysis of the clearance efficiency of clodronate liposomes on RAMs. (C) Quantification of the data in (B), n = 3. (D) Immunohistochemical staining for macrophage clearance (F4/80 positive) following clodronate liposome airway instillation. (E) Western blot analysis of PXR downstream genes Ugt1a1, GSTM2, and NRF2 protein levels in lung tissue after RAM depletion. (F) Semi-quantitative analysis of the Western blot data in (E). Data are presented as mean ± SEM. **p* < 0.05, ***p* < 0.01.


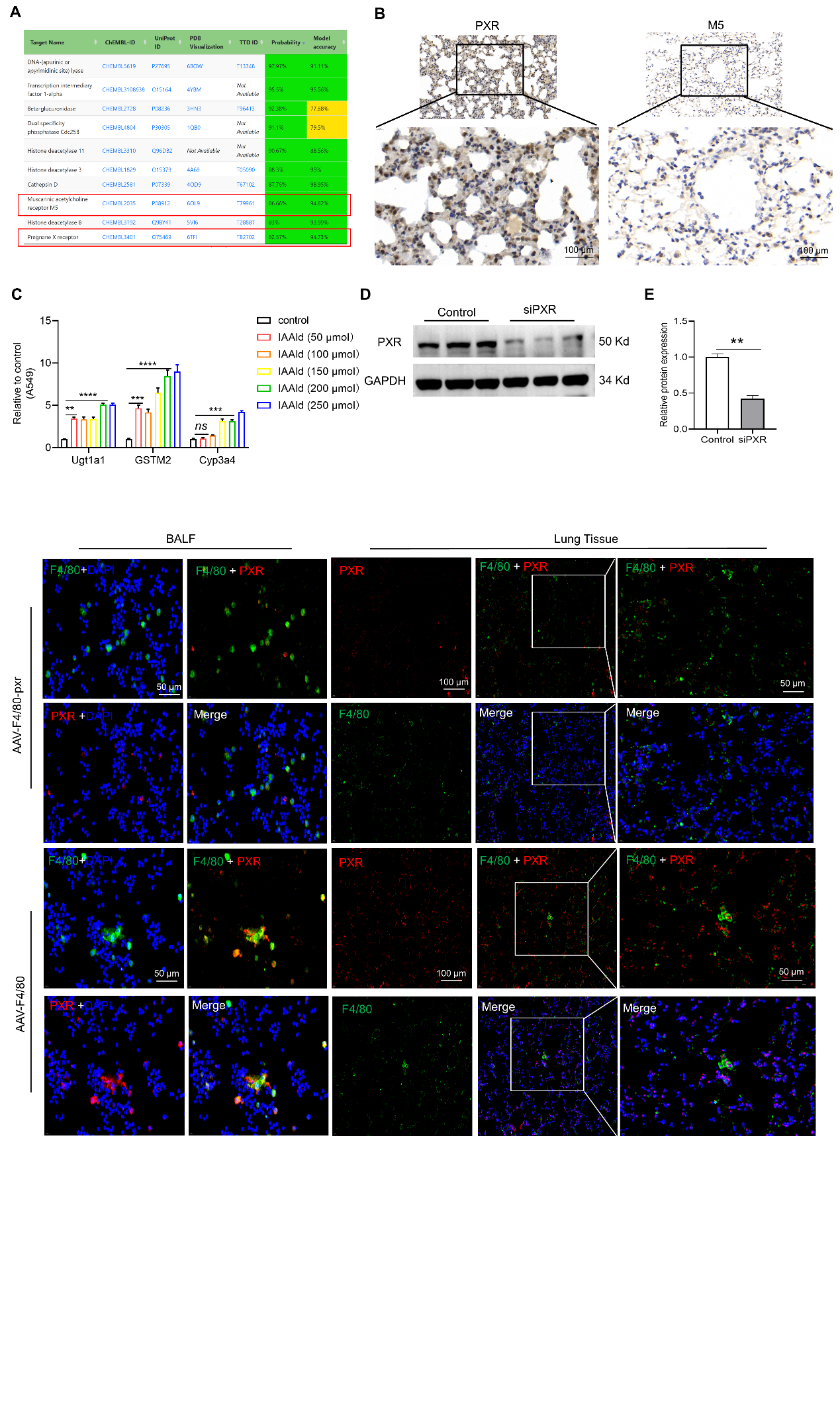


Figure S4. Prediction of IAAld receptors and their effects on gene activation. (A-B) Prediction of potential IAAld receptors using SuperPred and validation of their expression levels. (C) Verification of PXR downstream gene activation in human lung adenocarcinoma cell line A549 following IAAld stimulation. (D) Inhibition of PXR expression using small interfering RNA (siRNA), followed by Western blot analysis to confirm protein level reduction. (E) Semi-quantitative analysis of the Western blot data in (D). Data are presented as mean ± SEM. ***p* < 0.01, ****p* < 0.001, *****p* < 0.0001, *ns*: not significant.


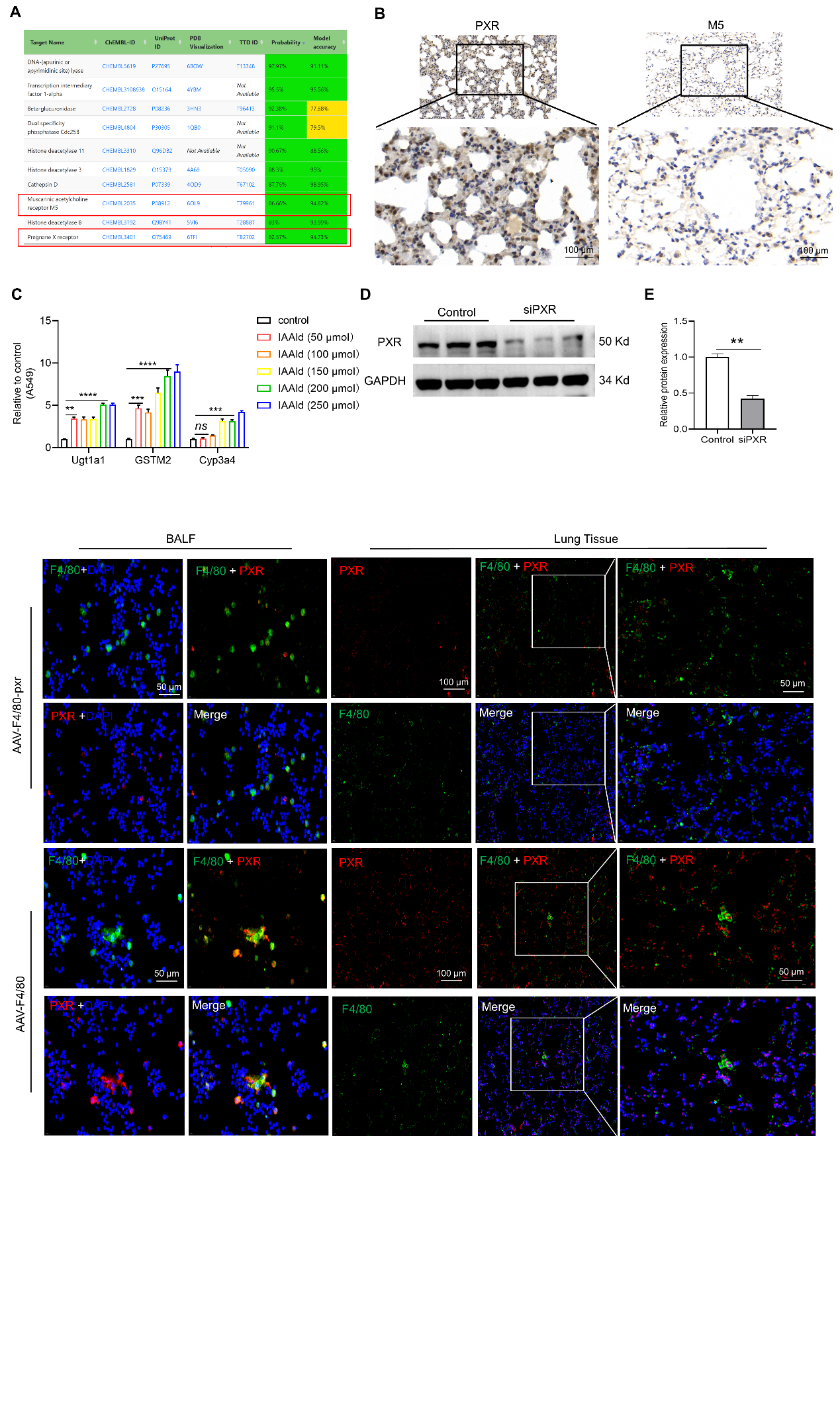


Figure S5. Validation of AAV-F4/80-pxr airway instillation for macrophage depletion in the lung. Images of cell from bronchoalveolar lavage fluid (BALF) of mice following AAV-F4/80-pxr intratracheally, with immunofluorescence staining. Green represents F4/80, red represents PXR, and blue indicates cell nuclei stained with DAPI (left panel). Right panel shows lung tissue from AAV-F4/80-pxr mice.


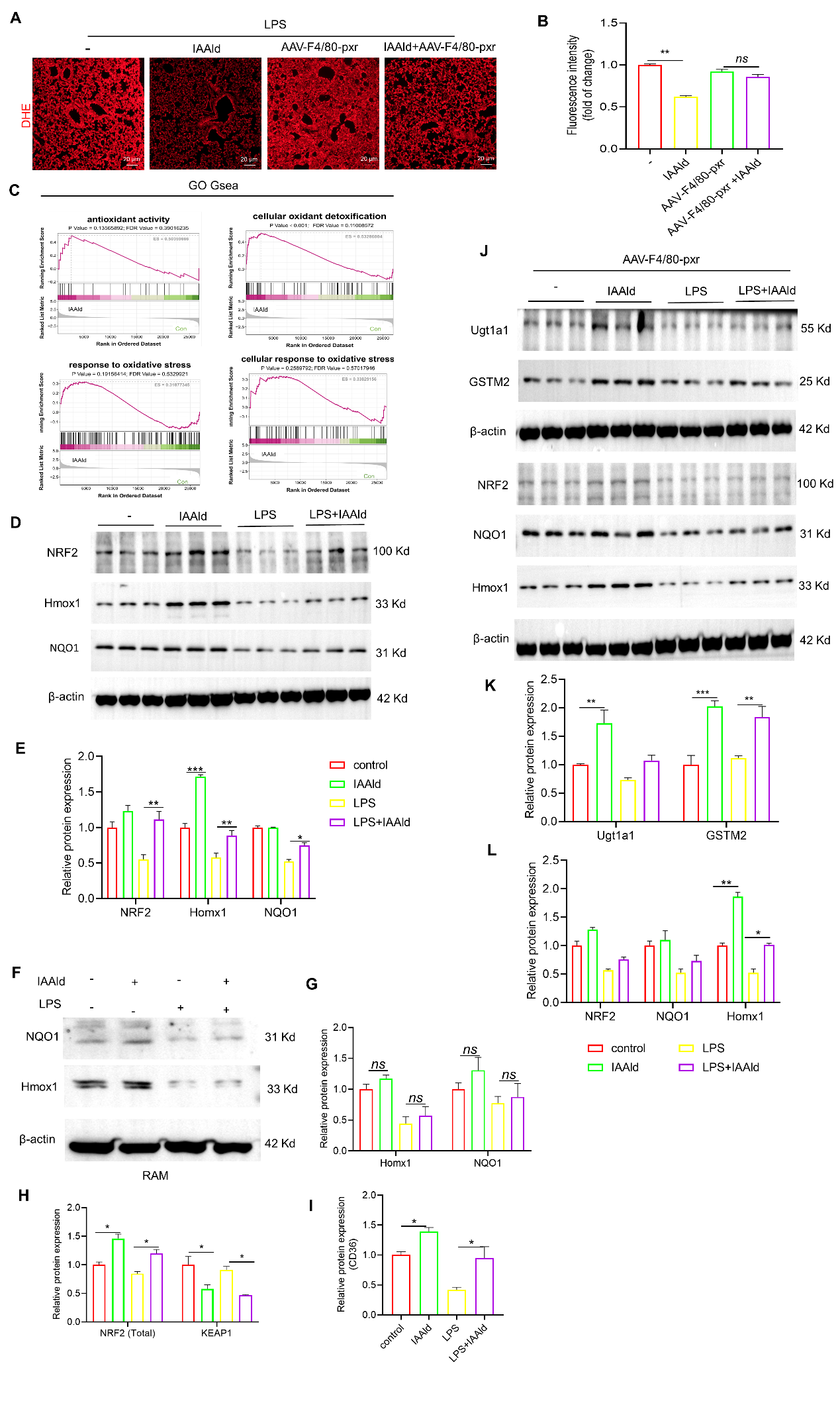


Figure S6. Modulation of oxidative stress by IAAld following LPS stimulation. (A) DHE staining to assess overall oxidative stress levels in mouse lung tissue, with semi-quantitative analysis shown in (B). (C) Gene Set Enrichment Analysis (GSEA) using the Gene Ontology (GO) database to evaluate oxidative stress-related pathways in MH-S cells after IAAld intervention. (D) Western blot analysis of NRF2 and antioxidant stress-related proteins Hmox1 and NQO1 levels in mouse lung tissue, with semi-quantitative analysis shown in (E). (F) Expression of antioxidant stress-related proteins in MH-S cells following IAAld treatment, with semi-quantitative analysis shown in (G). (H) Semi-quantitative analysis of data in Figure 7G. (J) Expression of related proteins in lung tissue following intratracheal administration of AAV-F4/80-pxr. (K–L) Semi-quantitative analysis of the protein expression shown in (J). Data are presented as mean ± SEM. **p* < 0.05, ***p* < 0.01, ****p* < 0.001, *ns*: not significant.


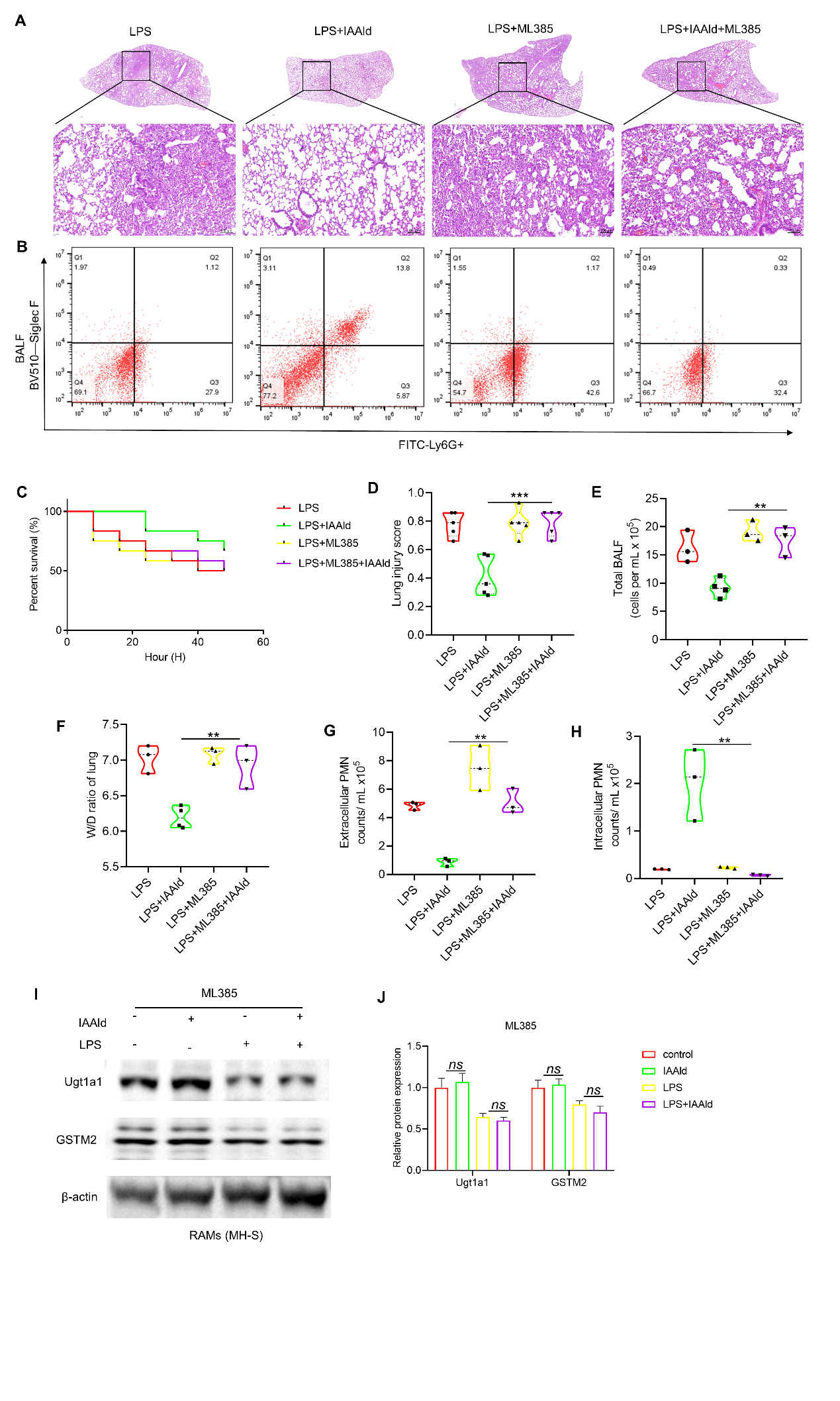


Figure S7. NRF2 inhibition counteracts the protective effects of IAAld in ARDS. ML385 (30 mg/kg) was intraperitoneally injected for 7 consecutive days before subsequent interventions. (A) Representative lung histological sections. (B) Flow cytometry analysis showing Siglec-F and Ly6G double-positive cells as RAM phagocytosing neutrophils (intracellular PMNs), and Ly6G+ cells as unphagocytosed neutrophils (extracellular PMNs). (C) Survival curves for ARDS mice following RAM PXR inhibition and IAAld treatment. (D) Histological damage scores, n = 5. (E) Cell counts in bronchoalveolar lavage fluid (BALF), n = 3~4. (F) Lung wet-to-dry weight ratio, n = 3~4. (G) and (H) Quantification of the flow cytometry data in (B), n = 3. (I) Evaluation of PXR downstream gene activation levels in RAMs after NRF2 inhibition, with semi-quantitative analysis shown in (J). Data are presented as mean ± SEM. ***p* < 0.01, ****p* < 0.001, *ns*: not significant.
